# *Helicobacter pylori* actively suppresses innate immune nucleic acid receptors

**DOI:** 10.1080/19490976.2022.2105102

**Published:** 2022-07-29

**Authors:** Samuel D.R. Dooyema, Jennifer M. Noto, Lydia E. Wroblewski, M. Blanca Piazuelo, Uma Krishna, Giovanni Suarez, Judith Romero-Gallo, Alberto G. Delgado, Richard M. Peek

**Affiliations:** a Department of Pathology, Microbiology, and Immunology, Vanderbilt University Medical Center, Nashville, Tennessee, USA; b Microbe-Host Interactions Training Program, Vanderbilt University, Nashville, Tennessee, USA; c Department of Medicine, Vanderbilt University Medical Center, Nashville, Tennessee, USA

**Keywords:** Helicobacter pylori, gastric cancer, TLR9, STING, RIG-I, TRIM30, innate immunity

## Abstract

Chronic mucosal pathogens have evolved multiple strategies to manipulate the host immune response; consequently, microbes contribute to the development of >2 million cases of cancer/year. Gastric adenocarcinoma is the fourth leading cause of cancer-related death and *Helicobacter pylori* confers the highest risk for this disease. Gastric innate immune effectors can either eliminate bacteria or mobilize adaptive immune responses including Toll-like receptors (TLRs), and cytosolic DNA sensor/adaptor proteins (e.g., stimulator of interferon genes, STING). The *H. pylori* strain-specific *cag* type IV secretion system (T4SS) augments gastric cancer risk and translocates DNA into epithelial cells where it activates the microbial DNA sensor TLR9 and suppresses injury *in vivo*; however, the ability of *H. pylori* to suppress additional nucleic acid PRRs within the context of chronic gastric inflammation and injury remains undefined. In this study, *in vitro* and *ex vivo* experiments identified a novel mechanism through which *H. pylori* actively suppresses STING and RIG-I signaling via downregulation of IRF3 activation. *In vivo*, the use of genetically deficient mice revealed that Th17 inflammatory responses are heightened following *H. pylori* infection within the context of *Sting* deficiency in conjunction with increased expression of a known host immune regulator, Trim30a. This novel mechanism of immune suppression by *H. pylori* is likely a critical component of a finely tuned rheostat that not only regulates the initial innate immune response, but also drives chronic gastric inflammation and injury.

## Introduction

Mucosal pathogens have evolved multiple strategies to manipulate the host immune response;^1,2^ consequently, microbes contribute to the development of greater than 2 million cases of cancer/year.^3^ Gastric adenocarcinoma is the fourth leading cause of cancer-related death^4–6^ and chronic infection with *Helicobacter pylori* confers the highest known risk for this disease.^3,6^ Initial components of the innate immune system encountered by *H. pylori* include epithelial cells, macrophages, and dendritic cells, and interactions between *H. pylori* and these constituents dysregulate signaling pathways that influence oncogenesis.^7,8^ Epithelial cells express effectors that can either eliminate bacteria or mobilize adaptive immune responses; these include pattern-recognition receptors (PRRs), which detect and respond to conserved microbial motifs.^9,10^ Functionally distinct PRR subclasses include Nod-like receptors (NLRs), Toll-like receptors (TLRs), and cytosolic DNA sensor/adaptor proteins (*e.g*., stimulator of interferon genes, STING), all of which are linked to gastric cancer.^10,11^ PRRs orchestrate immune responses targeting pathogens and bridge innate and adaptive immunity via recognition of pathogen-associated molecular patterns (PAMPs).^9,10^ However, *H. pylori* harbors multiple PAMPs that function differently than the respective counterparts in other mucosal pathogens. Specifically, 1) *H. pylori* FlaA (the major flagellin subunit) is a non-inflammatory molecule in terms of its ability to activate TLR5,^1^ 2) *H. pylori* LPS induces an attenuated TLR4-mediated response,^12,13^ 3) deacetylation of peptidoglycan allows *H. pylori* to evade host clearance via activation of a Nod1-dependent negative feedback loop,^14–17^ and 4) TLR9 suppresses the injury response to this pathogen.^18^ Thus, *H. pylori* has evolved an array of diverse phenotypes to subvert obstacles presented by the host, which promotes long-term colonization and carcinogenesis.

*H. pylori* strains exhibit a high level of genetic diversity^19,20^ and one strain-specific determinant that significantly augments cancer risk is the *cag* type IV secretion system (T4SS).^21–24^ The *cag* T4SS translocates a proinflammatory and oncogenic protein, CagA, as well as peptidoglycan and a metabolic intermediary in the LPS synthesis pathway, heptose bis-phosphate, into epithelial cells.^25–30^ Our laboratory has demonstrated that the *cag* T4SS also translocates microbial DNA, which subsequently activates TLR9.^18,31^ However, most persons colonized with *cag*^+^ strains do not develop cancer,^4,5,32^ suggesting that other *H. pylori* constituents also affect disease risk.

Microbial-specific nucleic acids are an important subclass of PAMPs, which are rapidly detected in the cytosol of host cells.^33,34^ Cyclic GMP-AMP synthase (cGAS) is a cytosolic DNA sensor, which is activated in response to double-stranded DNA in a sequence-independent manner. Binding of DNA ligands to cGAS catalyzes the conversion of ATP and GTP into the dinucleotide 2’,3’-cyclic GMP-AMP (cGAMP). cGAMP can then directly activate stimulator of interferon genes (STING), a DNA sensor/adaptor localized to the endoplasmic reticulum (ER)^33,34^ and which is expressed in gastric epithelial cells.^11^ Sensing of cyclic dinucleotides induces a conformational change in STING that triggers trafficking of STING complexed with TANK-binding kinase 1 (TBK1) from the ER to endosomal/lysosomal compartments. Translocated TBK1 leads to phosphorylation and activation of the transcription factor interferon regulatory factor 3 (IRF3), which is then mobilized to the nucleus to induce expression of type 1 interferons (*e.g*., IFNα, IFNß).^33,34^ STING activation can also trigger other downstream pathways such as NF-κB^35,36^ as well as autophagy, which clears DNA or pathogens from the cytosol.^37^

However, certain chronic pathogens have developed strategies to evade STING-mediated immune clearance, establish infection, and induce disease.^33,34,38^ Carcinogenic DNA viruses, such as, human papilloma virus (HPV) 18 and human adenovirus 5 encode the oncoproteins E7 and E1A, respectively, antagonize STING. Kaposi’s sarcoma-associated herpesvirus (KSHV) and hepatitis B express IRF1, tegument protein ORF52, and viral polymerases that potently disrupt the cGAS-STING pathway. Viral poxins abrogate STING signaling by degrading cGAMP.^33,34^ This wide repertoire of antagonists targeting cGAS-STING underscores the importance of evolutionary pressures that select for oncogenic pathogens that can both promote malignancy and suppress innate immunity.

The role of DNA sensing in human carcinogenesis is not fully understood but recent studies indicate that DNA sensors exert a crucial role in antitumor responses. Suppression of STING in prostate and melanoma cancer cells leads to increased tumor growth,^39,40^ and poor patient survival is associated with reduced cGAS and STING expression.^11,41^ In gastric cancer, STING expression is significantly decreased in tumor versus non-tumor tissue, and low levels of expression are associated with reduced survival.^11^ In models of inflammation with premalignant potential (*e.g*., chronic pancreatitis), inhibition of STING worsens disease via up-regulation of IL-17A,^42^ which promotes inflammation-induced malignancies including pancreatic cancer, colitis-associated carcinoma, and skin cancer.^43–45^

We previously demonstrated that translocated *H. pylori* DNA can activate the microbial DNA sensor TLR9 *in vitro* and that TLR9 suppresses *H. pylori*-induced injury *in vivo*; however, the ability of *H. pylori* to suppress additional nucleic acid PRRs within the context of gastric inflammation and injury has not been fully investigated. Therefore, the goal of this study was to elucidate the effects of *H. pylori* on STING signaling, and, using a *Sting*-deficient mouse model, delineate the role of STING in the context of gastric inflammation and injury. Our findings identify a novel mechanism through which *H. pylori* actively suppresses STING-associated signaling in host cells via induction of an induced host effector, Trim30a. These pathways may contribute to the ability of *H. pylori* to dampen the innate immune response and ultimately promote chronic gastric inflammation and injury, which may heighten the subsequent risk for gastric carcinogenesis.

## Results

**Figure 1.**
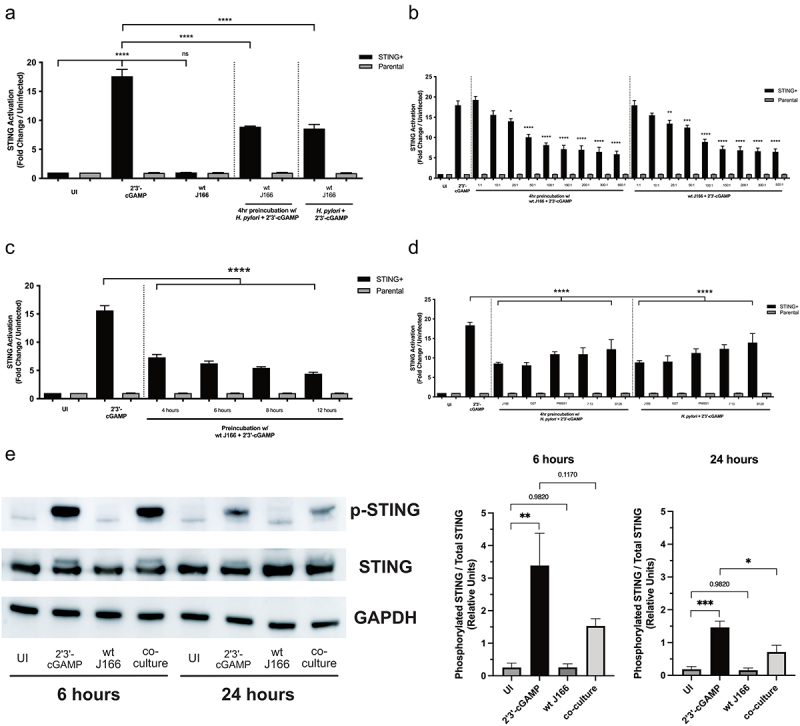
**STING activation is reduced by *H. pylori in vitro***. STING+ or parental cells were challenged with PBS alone (UI), 2ʹ3’-cGAMP and/or (a) wild-type (wt) *cag^+^ H. pylori* strain J166, (b) *H. pylori* strain J166 at varying MOIs, (c) increasing pre-incubation times, or (d) *cag^+^ H. pylori* strains J166, G27, PMSS1, 7.13, or B128 at MOI 100:1 for 24 hours. STING activation was assessed and is shown as fold STING activation relative to uninfected control. Experiments were performed in triplicate, and samples were run in duplicate within each experiment. ANOVA with Bonferroni correction was used to determine statistical significance among groups. (e) STING+ or parental cells were challenged with PBS alone (UI), 2ʹ3’-cGAMP and/or *H. pylori* strain J166 for 6 and 24 hours and were then assessed for STING expression and activation by Western blot analysis. For Western blot analysis, conditions were tested at least 3 times and student’s t-tests were used to determine statistical significance between groups. *p < .05, **p < .01, ***p < .001, ****p < .0001, ns = not significant.

We first sought to directly assess the effects of *H. pylori* on STING signaling, by utilizing HEK293 cells transfected with a STING-specific reporter. While levels of STING activation increased 17-fold in cells co-cultured with 2ʹ3’-cGAMP (a STING agonist), activation levels in cells co-cultured with the wild-type *cag^+^ H. pylori* strain J166 were no different than uninfected controls (Figure 1(a)). Because certain chronic pathogens have been shown to exert a suppressive effect on STING signaling and to facilitate long term survival, we next simultaneously co-cultured or pre-incubated cells with *H. pylori* prior to the addition of 2ʹ3’-cGAMP. *H. pylori* significantly reduced STING agonist-mediated activation by 50% in both condition (Figure 1(a)). STING suppression by *H. pylori* was dose- (Figure 1(b)) and time-dependent (Figure 1(c)) with minimal effects on cell viability out to 16–24 hours (Supplemental Figure 1). Previous work defining the ability of *H. pylori* to activate the innate immune DNA sensor TLR9 utilized strain J166.^31^ Therefore, to determine if STING suppression was strain-specific, we repeated these studies using additional isolates and demonstrated that *H. pylori* strain G27, the rodent-colonizing strains PMSS1 and 7.13, and clinical isolate B128 all significantly reduced STING activation under either preincubation or co-culture conditions compared to agonist alone (Figure 1(d)). To assess whether reduced activation was attributable to reduced STING expression, we assessed STING expression and activation (pSTING) in HEK293 cells treated with the STING agonist (2ʹ3’-cGAMP) with or without *H. pylori* strain J166 (Figure 1(e)). These data demonstrate that HEK293 cells express similar levels of STING within all treatment groups. Similar to the STING reporter assay, activation of STING (pSTING) is significantly increased with the STING agonist (2ʹ3’-cGAMP), while *H. pylori* significantly reduced STING agonist-mediated activation by 50% (Figure 1(e)). To determine whether 2ʹ3’-cGAMP directly altered bacterial growth or function *per se, H. pylori* was cultured in the absence of eukaryotic cells with varying concentrations of 2ʹ3’-cGAMP. Treatment of *H. pylori* with 2ʹ3’-cGAMP had no effect on *H. pylori* growth or the ability of *H. pylori* to translocate CagA into host cells (Supplemental Figure 2).

**Figure 2.**
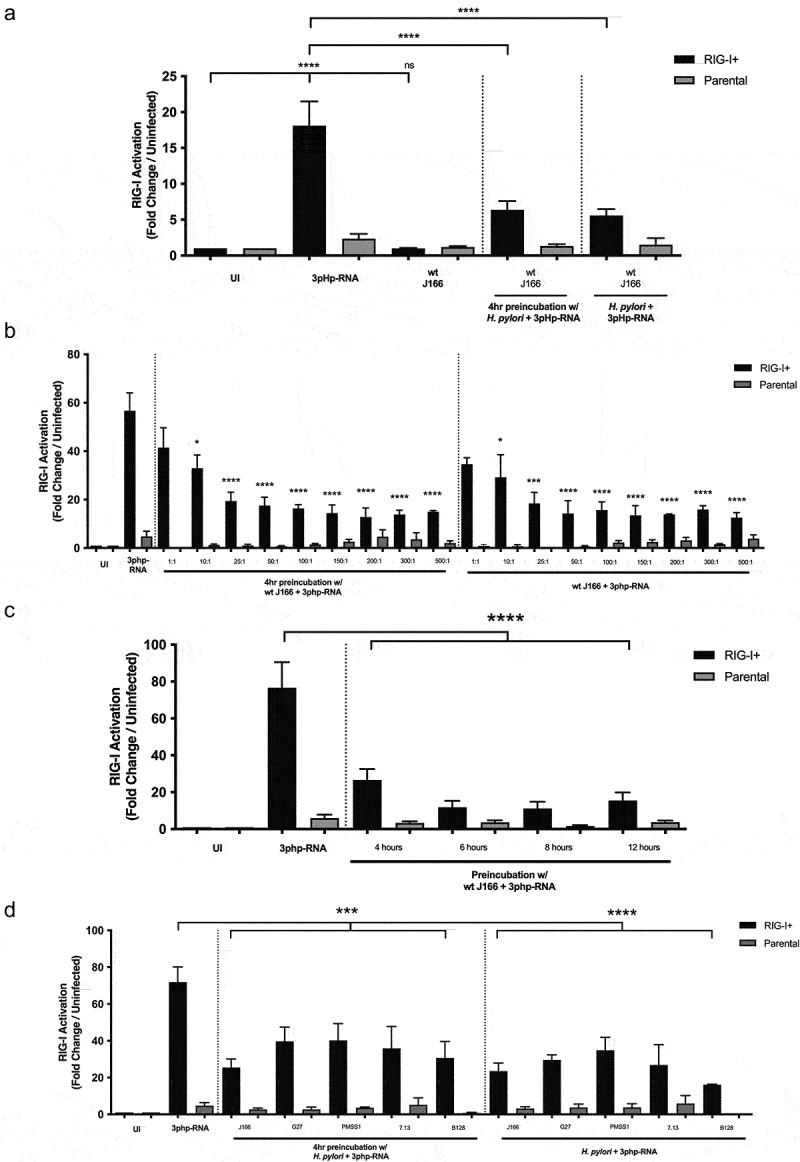
**RIG-I activation is attenuated by *H. pylori in vitro***. RIG-I+ or parental cells were challenged with PBS alone (UI), RIG-I agonist 3p-hpRNA and/or (a) wild-type (wt) *cag^+^ H. pylori* strain J166 at MOI 100:1 for 24 hours, (b) *H. pylori* strain J166 at varying MOIs, (c) increasing pre-incubation times, or (d) *cag^+^ H. pylori* strains J166, G27, PMSS1, 7.13, or B128. RIG-I activation was assessed and is shown as fold RIG-I activation relative to uninfected control. Experiments were performed in triplicate, and samples were run in duplicate within each experiment. ANOVA with Bonferroni correction was used to determine statistical significance among groups. *p < .05, ***p < .001, ****p < .0001, ns = not significant.

Based on the premise that translocated microbial DNA may be transcribed in host cells and subsequently activate RNA sensing molecules as previously reported in *Legionella pneumophila*,^46,47^ the ability of *H. pylori* to suppress activation of RIG-I, an RNA sensor, was also investigated. In HEK293 cells transfected with a RIG-I-specific reporter, RIG-I-associated activation increased 18-fold in cells co-cultured with 3pHp-RNA, a known RIG-I agonist, compared to untreated controls (Figure 2(a)). Similar to results observed with STING activation, no RIG-I activation was observed in cells co-cultured with *H. pylori* alone (Figure 2(a)). However, a suppression phenotype was observed during pre-incubation with *H. pylori* prior to addition of 3pHp-RNA or during co-culture of *H. pylori* and agonist together (Figure 2(a)), and this occurred in a dose- (Figure 2(b)) and time-dependent (Figure 2(c)) manner. Cell viability of RIG-I reporter cells was not significantly affected by the presence of *H. pylori* and/or agonist (Supplemental Figure 3). Mirroring the STING results, multiple *H. pylori* strains were able to significantly attenuate RIG-I-associated signaling *in vitro* (Figure 2(d)).

**Figure 3.**
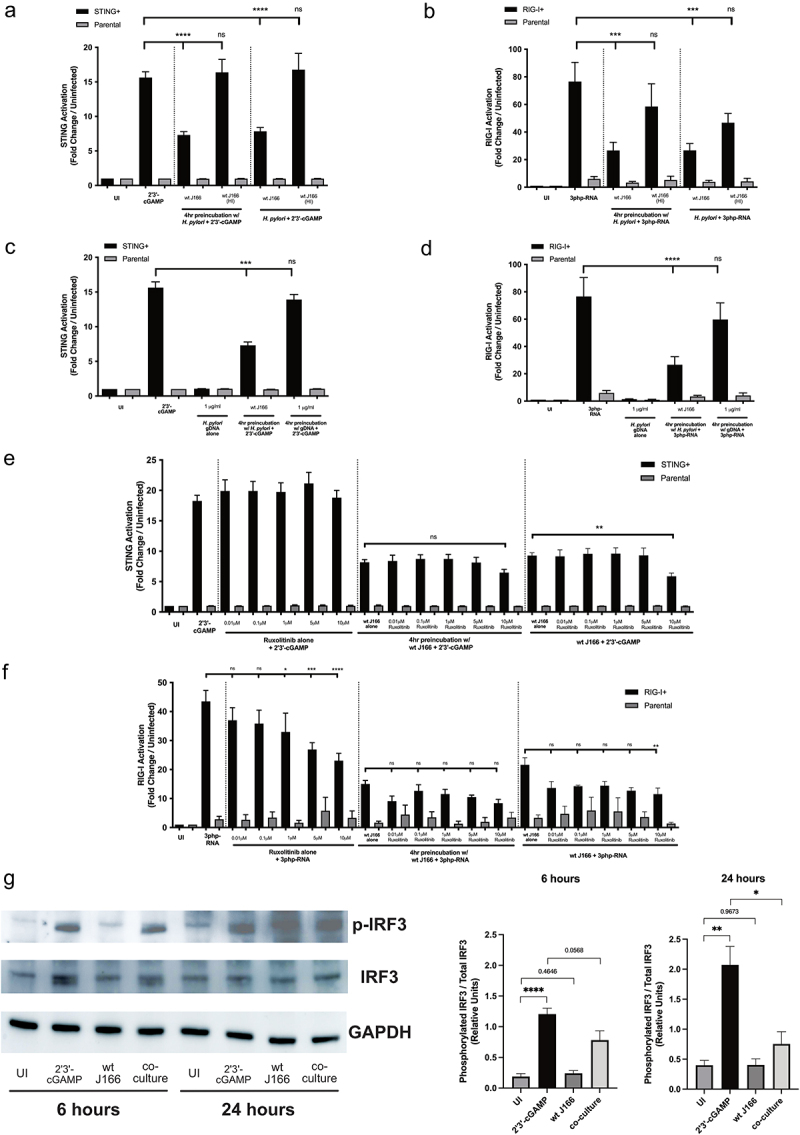
***H. pylori* suppresses STING and RIG-I activation *in vitro***. (a) STING+ or parental cells were challenged with PBS alone (UI), 2ʹ3’-cGAMP and/or viable or heat inactivated (HI) wild-type (wt) *H. pylori* strain J166. (b) RIG-I+ or parental cells were challenged with PBS alone (UI), 3p-hpRNA and/or viable or heat inactivated (HI) *H. pylori* strain J166. (c) STING+ or parental cells were challenged with PBS alone (UI), 2ʹ3’-cGAMP, and/or *H. pylori* strain J166 or *H. pylori* gDNA. (d) RIG-I+ or parental cells were challenged with PBS alone (UI), 3p-hpRNA with or without *H. pylori* strain J166 or *H. pylori* gDNA. (e) STING+ or parental cells were challenged with PBS alone (UI), 2ʹ3’-cGAMP, and/or *H. pylori* strain J166 in the presence of increasing concentrations of Ruxolitinib. (f) RIG-I+ or parental cells were challenged with PBS alone (UI), 3p-hpRNA and/or *H. pylori* strain J166 in the presence of increasing concentrations of Ruxolitinib. STING and RIG-I activation was assessed and data are shown as fold activation relative to uninfected control. Experiments were performed in triplicate, and samples were run in duplicate within each experiment. ANOVA with Bonferroni correction was used to determine statistical significance between groups. (g) STING+ or parental cells were challenged with PBS alone (UI), 2ʹ3’-cGAMP with or without *H. pylori* strain J166 for 6 and 24 hours and were then assessed for IRF3 expression and activation by Western blot analysis. For Western blot analysis, conditions were tested at least 3 times and student’s t-tests were used to determine statistical significance between groups. *p < .05, **p < .01, ***p < .001, ****p < .0001, ns = not significant.

To determine whether the suppression phenotype for STING and RIG-I required an active interplay between *H. pylori* and host cells, we repeated the STING and RIG-I reporter assay experiments with wild-type *H. pylori* J166 that had been heat-inactivated for 1 hour at 56°C. Heat-inactivation abolished the suppressive phenotype for both STING and RIG-I (Figures 3(a-b)). Further, *H. pylori* genomic DNA *per se* was unable to suppress STING- and RIG-I-associated signaling *in vitro* when compared to viable *H. pylori* (Figure 3(c-d)). These results demonstrate that only viable *H. pylori* can suppress STING- and RIG-I-associated signaling *in vitro*.

The InvivoGen HEK-Blue™ hSTING-R232 cells and HEK-Lucia™ RIG-I cells used for these assays are predominantly dependent on activation of IRF3 which, when phosphorylated, induces type I interferon (IFN) gene expression. The IFN-stimulated response elements (ISRE) luciferase reporter in RIG-I reporter cells, however, can also be activated by type I IFN activation which is induced by JAK/STAT signaling through ISG54. Therefore, to determine whether the observed RIG-I phenotype was IRF3- versus JAK/STAT-dependent, we utilized the inhibitor Ruxolitinib, which inhibits downstream type I IFN signaling via the JAK-STAT pathway, leaving IRF3-dependent signaling unaffected. Ruxolitinib in the presence of *H. pylori* and positive agonists failed to alter suppression when compared to *H. pylori* and agonist alone (Figure 3(e-f)). However, when Ruxolitinib and agonist alone were co-cultured without *H. pylori*, RIG-I-associated signaling but not STING-associated signaling was significantly reduced compared to the agonist alone (Figure 3(e-f)). These data suggest that *H. pylori* likely exerts inhibitory effects at the level of IRF in these signaling pathways. To more directly assess this, we next quantified IRF3 expression and activation (pIRF3) in HEK293 cells treated with the STING agonist (2ʹ3’-cGAMP) with or without *H. pylori* strain J166. These data demonstrate that the STING agonist (2ʹ3’-cGAMP) significantly increased IRF3 activation, and that this activation is significantly reduced during *H. pylori* co-culture (Figure 3(g)), indicating that *H. pylori* inhibits STING agonist-mediated IRF3 activation.

**Figure 4.**
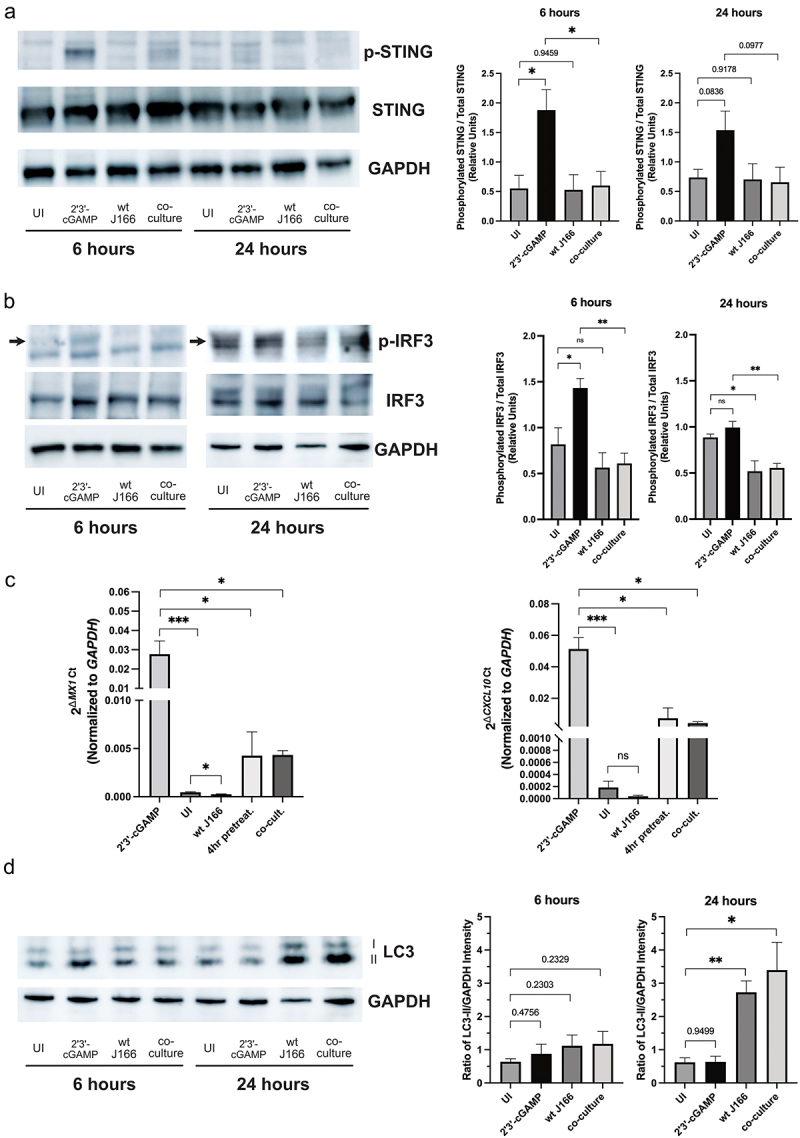
***H. pylori* downregulates STING and IRF3 activation but induces autophagy in human gastric organoids**. Human gastric organoid monolayers were challenged with PBS alone (UI), 2ʹ3’-cGAMP with or without wild-type (wt) *H. pylori* strain J166 at MOI 100:1 for 6 and 24 hours. (a) STING activation was determined by quantifying levels of phosphorylated STING (pSTING). (b) IRF3 activation was determined by quantifying levels of phosphorylated IRF3 (p-IRF3, arrow) in gastric organoid co-cultures. Representative Western blots and densitometric analyses normalizing levels of pSTING and pIRF3 to total levels are shown at each time point. (c) RT-PCR analysis of *MX1* and *CXCL10* transcript levels are represented as relative gene expression levels normalized to levels of *GAPDH* gene expression. (d) Induction of autophagy was determined by quantifying levels of LC3-II. Representative Western blots and densitometric analyses normalizing levels of LC3-II to GAPDH are shown at each time point. In each experiment, conditions were tested at least 3 times and student’s t-tests were used to determine statistical significance between groups. *p < .05, **p < .01, ***p < .001, ns = not significant.

Gastroids are polarized, replenishable epithelial culture systems that can be readily generated from non-transformed gastric epithelium.^48,49^ We previously developed and optimized gastroid models of *H. pylori* infection originating from both human and murine gastric tissues;^17,50^ therefore, we capitalized on this manipulatable *ex vivo* system as a biologically relevant model that more faithfully recapitulates the gastric niche to extend our *in vitro* results using reporter systems. Primary gastric organoids generated from human patients were co-cultured for 6 or 24 hours with wild-type *H. pylori* strain J166, with or without the STING agonist, and protein lysates were subsequently probed for STING activation and downstream effectors of STING activation via Western blot analysis. To directly assess STING activation, we quantified STING expression and activation (pSTING) in human gastric organoids treated with the STING agonist (2ʹ3’-cGAMP) with or without *H. pylori* strain J166. These data demonstrate that *H. pylori* significantly reduced STING agonist-mediated activation, but not expression, by 3-fold, (Figure 4(a)) consistent with the results from HEK293 cells (Figure 1(e)). IRF3, an effector activated by STING, was next assessed. *H. pylori*-infected human organoids harbored significantly lower levels of phosphorylated IRF3, compared to uninfected controls at both 6 and 24 hours (Figure 4(b)). The STING agonist, 2ʹ3’-cGAMP, alone induced significantly higher levels of pIRF3 at 6 hours compared to controls, but this did not occur during co-culture with *H. pylori*; this effect diminished by 24 hours (Figure 4(b)). Concordantly, expression levels of the IRF3-dependent type I interferon stimulated genes *MX1* and *CXCL10* were significantly upregulated in human gastric organoids following co-culture with 2ʹ3’-cGAMP (Figure 4(c)), but expression was significantly reduced in samples co-cultured with *H. pylori* and 2ʹ3’-cGAMP (Figure 4(c)). These results indicate that *H. pylori* infection of organoids recapitulated the suppressive phenotype observed in reporter cell assays (Figure 1).

STING complexes can recruit effectors which drive autophagy-like responses, that are independent of IRF3-mediated *Ifnb* transcription.^51^ *H. pylori* can also induce autophagy. Therefore, we analyzed autophagy under these conditions. All *H. pylori*-infected samples demonstrated a significant increase in levels of autophagy compared to uninfected samples by 24 hours when utilizing increases in the autophagosome marker LC3-II as a proxy for autophagy (Figure 4(d)). Collectively, these results suggest that *H. pylori* can suppress STING signaling via IRF3 and that *H. pylori*-induced autophagy is independent of STING.^52,53^

**Figure 5.**
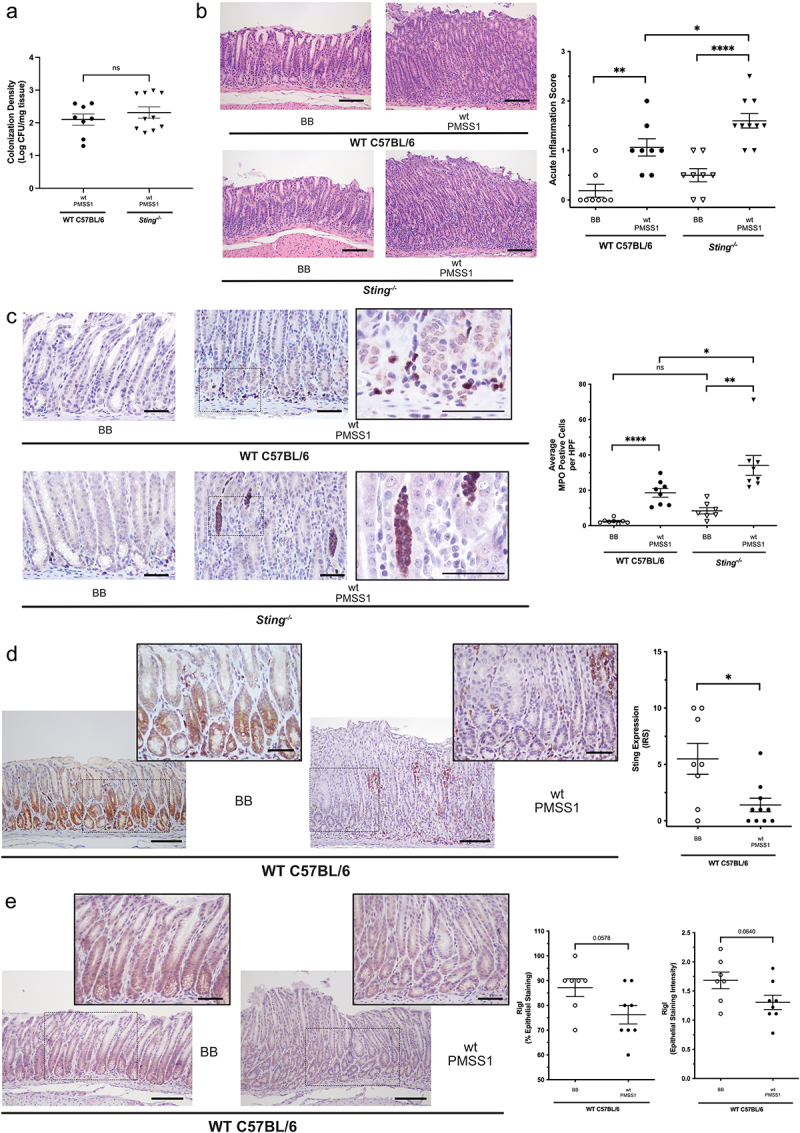
***H. pylori* infection significantly augments acute immune responses in *Sting*-deficient mice and decreases Sting and RigI expression in wild-type mice**. Wild-type (WT) C57BL/6 and *Sting^−/−^* mice were challenged with Brucella broth (BB) or wild-type (wt) *H. pylori* strain PMSS1 for 8 weeks. (a) Gastric sections were homogenized and serially diluted on blood-agar plates to quantify *H. pylori* colonization in infected mice. (b) Acute inflammation was assessed and scored in the antrum and corpus of C57BL/6 wild-type or *Sting^−/−^* mice infected with or without *H. pylori* by a pathologist blinded to treatment groups. Histologic parameters were scored according to the Sydney System.^54^ Representative images of acute inflammation are shown at 200x. Scale bars = 100 µm. (c) MPO was assessed by IHC in wild-type or *Sting^−/−^* mice infected with or without *H. pylori*. Representative images of antral MPO IHC are shown at 400x and 1000x magnification. Scale bars = 50 µm. MPO^+^ cells were enumerated in 5 high-powered fields (HPF) from each mouse and averaged. Sting (d) and RigI (e) were assessed by IHC in wild-type mice infected with or without *H. pylori*. Representative images of antral Sting and RigI IHC are shown at 200x and 400x magnification. Scale bars = 100 µm (200x) and 50 µm (400x). (D) Sting immunoreactive score (IRS) gives a range of 0–12 as a product of multiplication between positive cells proportion score (0–4) and staining intensity score (0–3) across 5 HPFs from each animal. (E) RigI staining is shown as percent epithelial staining and epithelial staining intensity. Each data point represents an individual animal (WT BB, n = 8; WT PMSS1, n = 8; *Sting^−/−^* BB, n = 8; *Sting^−/−^* PMSS1, n = 10) from one experiment. Student’s t-tests were used to determine statistical significance between groups. *p < .05, **p < .01, ***p < .001, ****p < .0001, ns = not significant.

We next extended these findings by defining the role of STING in *H. pylori*-induced injury *in vivo*. Wild-type C57BL/6 and *Sting*-deficient mice (*Sting*^−/−^) were infected with the *cag^+^ H. pylori* strain PMSS1, which harbors the ability to suppress STING-associated signaling *in vitro* (Figure 1(d)). No significant differences in levels of *H. pylori* colonization were present between wild-type and *Sting*^−/−^ mice following 8 weeks of infection (Figure 5(a)). As expected, wild-type mice infected with *H. pylori* developed significantly increased levels of acute and chronic inflammation compared to uninfected controls (Figure 5(b), Supplemental Figure 4). However, *Sting* genetic deficiency augmented acute (Figure 5(b)), but not chronic inflammation (Supplemental Figure 4), compared to infected wild-type mice. Further immunophenotyping by immunohistochemistry staining revealed significantly higher levels of neutrophils among infected *Sting*^−/−^ mice compared to infected wild-type mice (Figure 5(c)), while the number of macrophages, T cells, and B cells remained unchanged regardless of *Sting* status (Supplemental Figure 5), suggesting that STING alters acute inflammatory events during *H. pylori* infection.

Decreased STING expression has been shown to be an independent and adverse predictor of overall survival in human gastric cancer patients.^11^ Examination of gastric tissue from wild-type mice by immunohistochemistry revealed that levels of Sting expression among *H. pylori*-infected gastric tissue were significantly reduced compared to uninfected tissues (Figure 5(d)) and, as expected, undetectable in *Sting*^−/−^ mice (data not shown). Furthermore, levels of RigI were also markedly reduced in *H. pylori*-infected gastric tissue compared to uninfected wild-type mice (Figure 5(e)). These data indicate that *H. pylori* can not only suppress Sting activation and signaling, but can also reduce levels of Sting and RigI expression *in vivo*.

**Figure 6.**
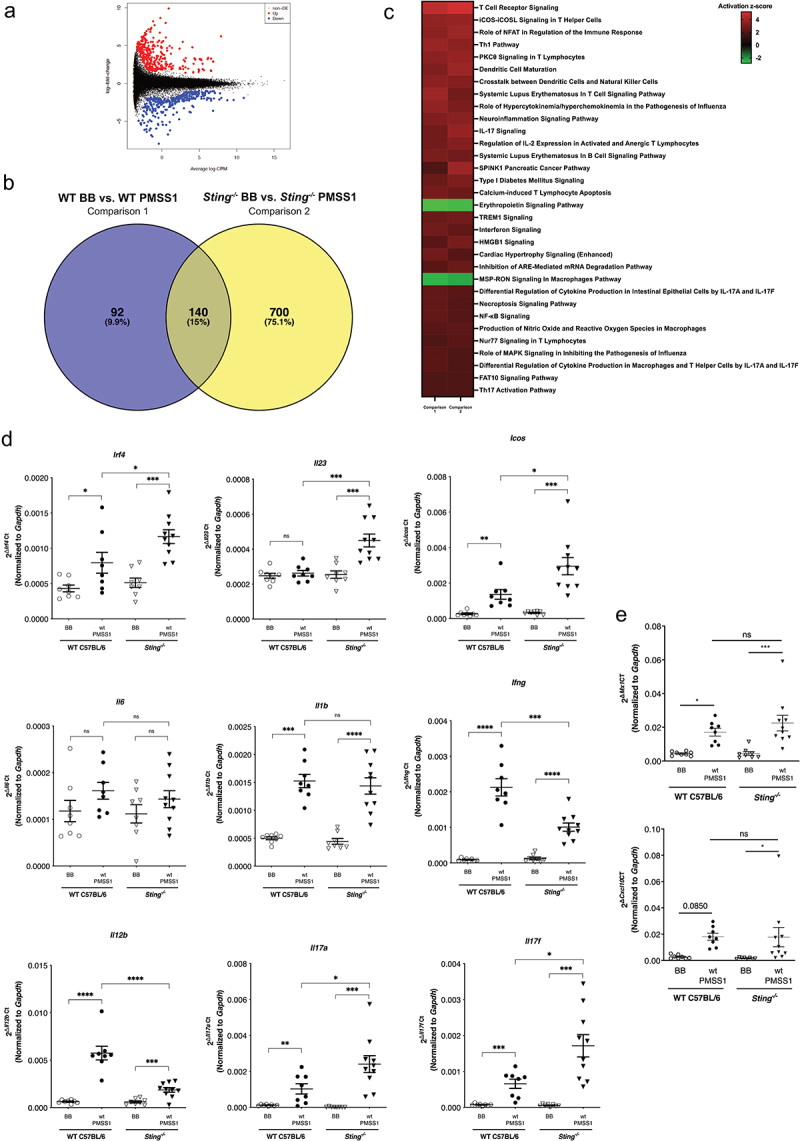
**Differential gene expression between uninfected and infected wild-type C57BL/6 and *Sting^−/−^* mice from RNA-seq data**. (a) Volcano plot representing differentially expressed genes in the RNA-seq dataset of uninfected wild-type (WT) C57BL/6 and *Sting^−/−^* mice at baseline. (b) Venn diagram representing differentially expressed genes in the RNA-seq dataset of uninfected and *H. pylori*-infected wild-type (WT) C57BL/6 and *Sting^−/−^* mice. (c) Top significantly affected (2.0 < Z score < −2.0) canonical pathways based on Ingenuity Pathway Analysis (IPA). The horizontal bars denote the different pathways based on the Z-scores. Red indicates activation, while green indicates suppression. (d) mRNA expression of Th17-related genes in uninfected and *H. pylori-*infected wild-type mice, and uninfected and *H. pylori*-infected *Sting^−/−^* mice. (e) mRNA expression of IRF3-dependent type I interferon stimulated genes, *Mx1* and *Cxcl10*, in uninfected and *H. pylori-*infected wild-type mice, and uninfected and *H. pylori*-infected *Sting^−/−^* mice. Data are represented as relative gene expression normalized to levels of *Gapdh* gene expression. Each data point represents an individual animal (WT BB, n = 8; WT PMSS1, n = 8; *Sting^−/−^* BB, n = 8; *Sting^−/−^* PMSS1, n = 10) from one experiment. Student’s t-tests were used to determine statistical significance between groups. *p < .05, **p < .01, ***p < .001, ****p < .0001, ns = not significant.

To identify specific effectors mediating suppression of Sting signaling by *H. pylori in vivo*, we next performed a discovery-based RNA-seq analysis utilizing RNA isolated from whole gastric mucosa from wild-type and *Sting*^−/−^ mice. Comparisons were performed to identify *Sting*-dependent responses at baseline and following *H. pylori* challenge. To first identify genes differentially expressed at baseline, datasets from uninfected wild-type and *Sting*^−/−^ mice were compared (Figure 6(a)). This comparison revealed that 336 upregulated and 325 downregulated genes were altered in a *Sting-*dependent manner (Supplemental Table 1). Next, to identify genes differentially expressed following *H. pylori* infection, datasets from uninfected versus infected wild-type mice were termed comparison 1, while uninfected versus infected *Sting*^−/−^ mice were termed comparison 2 (Figure 6(b)). Comparison 1 revealed 213 upregulated and 19 downregulated genes following *H. pylori* infection (Supplemental Table 2), while comparison 2 identified 840 genes differentially expressed in *Sting*^−/−^ mice following *H. pylori* infection, with 382 upregulated and 458 downregulated genes (Supplemental Table 3). Ingenuity Pathway Analysis software was then used to harmonize the datasets to identify predicted biological functions and pathways to reveal possible mechanisms that may underpin the suppressive phenotypes (Table 1).

**Table 1.** Top significantly affected (2.0 < Z score < −2.0) canonical pathways based on ingenuity pathway analysis between comparison 1 and comparison 2.

Pathway	1	2
T Cell Receptor Signaling	4.747	5.209
iCOS-iCOSL Signaling in T Helper Cells	3.606	3.742
Role of NFAT in Regulation of the Immune Response	3.464	3.873
Th1 Pathway	3.771	3.5
PKCθ Signaling in T Lymphocytes	3.464	3.742
Dendritic Cell Maturation	3.207	3.873
Crosstalk between Dendritic Cells and Natural Killer Cells	3.464	3.317
Systemic Lupus Erythematosus in T Cell Signaling Pathway	3.873	2.828
Role of Hypercytokinemia/hyperchemokinemia in the Pathogenesis of Influenza	3.606	3.051
Neuroinflammation Signaling Pathway	3.207	3.441
IL-17 Signaling	2.828	3.742
Regulation of IL-2 Expression in Activated and Anergic T Lymphocytes	2.828	3.317
Systemic Lupus Erythematosus In B Cell Signaling Pathway	3.051	2.84
SPINK1 Pancreatic Cancer Pathway	2	3.873
Type I Diabetes Mellitus Signaling	2.646	3.162
Calcium-induced T Lymphocyte Apoptosis	3	2.714
Erythropoietin Signaling Pathway	−2.828	−2.714
TREM1 Signaling	2.646	2.828
Interferon Signaling	2.828	2.449
HMGB1 Signaling	2.236	3
Cardiac Hypertrophy Signaling (Enhanced)	2.828	2.183
Inhibition of ARE-Mediated mRNA Degradation Pathway	2.236	2.646
MSP-RON Signaling In Macrophages Pathway	−2.53	−2.309
Differential Regulation of Cytokine Production in Intestinal Epithelial Cells by IL-17A and IL-17 F	2.449	2.236
Necroptosis Signaling Pathway	2.449	2.121
NF-κB Signaling	2.449	2.121
Production of Nitric Oxide and Reactive Oxygen Species in Macrophages	2.236	2.333
Nur77 Signaling in T Lymphocytes	2	2.236
Role of MAPK Signaling in Inhibiting the Pathogenesis of Influenza	2.236	2
Differential Regulation of Cytokine Production in Macrophages and T Helper Cells by IL-17A and IL-17 F	2.236	2
FAT10 Signaling Pathway	2	2
Th17 Activation Pathway	2	2

Pathway analysis revealed that predicted functions related to IL-17 signaling had significant activation scores (>2) in both wild-type and *Sting*^−/−^ mice (Figure 6(c)), consistent with prior data demonstrating the ability of *H. pylori* to induce IL-17 production and Th17 responses^17,18,55–57^ in conjunction with observations that inhibition of STING activation is associated with increased Th17 cell infiltration, increased production of IL-17A, and worsening inflammation in conditions such as chronic pancreatitis.^45^ Therefore, to independently validate potential differences in IL-17 signaling due to *Sting* deficiency within the context of *H. pylori* infection, we examined Th17 differentiation and stabilization factors via RT-PCR from RNA isolated from gastric tissue. Differentiation and formation of Th17 cells can be driven by transcription factors such as IRF4 and cytokines such as IL-23. Inducible Costimulator (ICOS) has also been shown to be critical for the development of Th17 cells. Transcript levels of *Irf4, Il23*, and *Icos* in *H. pylori*-infected *Sting^−/−^* mice were significantly increased compared to infected wild-type mice (Figure 6(d)), while no significant differences were observed in levels of *Il6* and *Il1b* (Figure 6(d)) suggesting that, in the absence of *Sting*, Th17 differentiation may be driven by both IL-23 and Icos, among other factors that were not evaluated. Conversely, significantly lower levels of the Th17 inhibitors *Ifng* and *Il12b* were present among infected *Sting*-deficient mice (Figure 6(d)). Transcript levels of *Il17a* and *Il17f* were also significantly increased in *H. pylori*-infected *Sting^−/−^* mice compared to infected wild-type mice (Figure 6(d)). In assessing IRF3-dependent type I interferon stimulated genes by RT-PCR, *Mx1* and *Cxcl10* were altered to a similar degree with *H. pylori* infection in both wild-type and *Sting^−/−^* mice (Figure 6(e)), which validate data obtained from RNA-seq.

**Figure 7.**
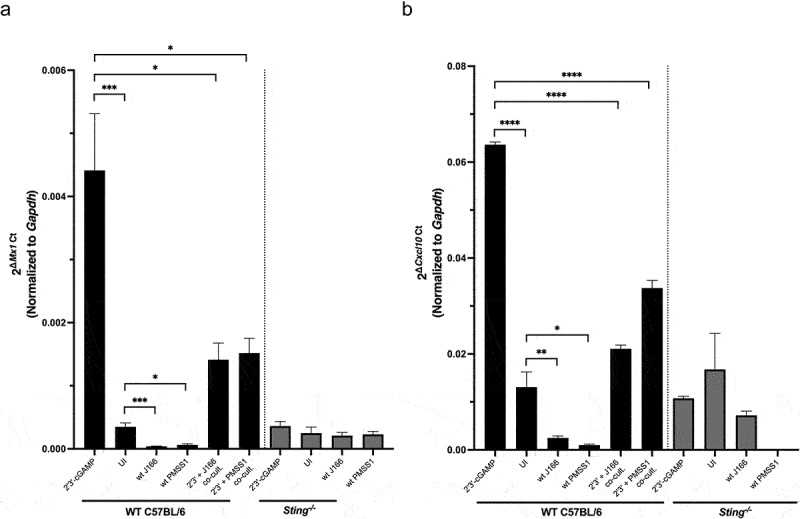
***H. pylori* infection of murine gastric organoids downregulates IRF3-dependent type I interferon stimulated genes**. Murine gastric organoid monolayers were challenged with PBS alone (UI), STING agonist 2ʹ3’-cGAMP, and/or wild-type (wt) *H. pylori* strain J166 or PMSS1 at MOI 100:1 for 6 or 24 hours. RT-PCR analysis of (a) *Mx1* and (b) *Cxcl10* transcript levels was assessed in co-cultured murine gastric organoids. Data are represented as relative gene expression levels normalized to levels of *Gapdh* gene expression. In each experiment, conditions were tested at least 3 times and student’s t-tests were used to determine statistical significance between groups. *p < .05, **p < .01, ***p < .001, ****p < .0001.

We next sought to recapitulate our human *ex vivo* findings (Figure 4) demonstrating that *H. pylori* infection can selectively downregulate phosphorylated-IRF3-dependent pathways in gastric epithelial cells and further delineate the role of *H. pylori* in regulating phenotypes linked to *Sting* deficiency within our murine model systems. Murine gastroid organoid monolayers were infected *ex vivo* with *H. pylori* strains J166 or PMSS1 for 24 hours and were used to analyze Sting downstream pathways via RT-PCR. IRF3-dependent type I interferon stimulated genes *Mx1* and *Cxcl10* were significantly upregulated in wild-type murine gastric organoids following co-culture with 2ʹ3’-cGAMP, but expression was significantly reduced in samples co-infected with *H. pylori* and 2ʹ3’-cGAMP, and no changes were observed in *Sting^−/−^* monolayers (Figure 7(a-b)). Collectively, these data illustrate the differences between *in vivo* and *ex vivo* models in terms of the microenvironment, as the RNA-seq data generated from mouse tissue and RT-PCR analysis from mouse and human gastric organoids show differential regulation of *Mx1* and *Cxcl10*. We speculate that in isolated organoid models, which lack immune components and other elements of the gastric microenvironment, differential responses in *Sting*-dependent regulation of *Mx1* and *Cxcl10* may occur.

**Figure 8.**
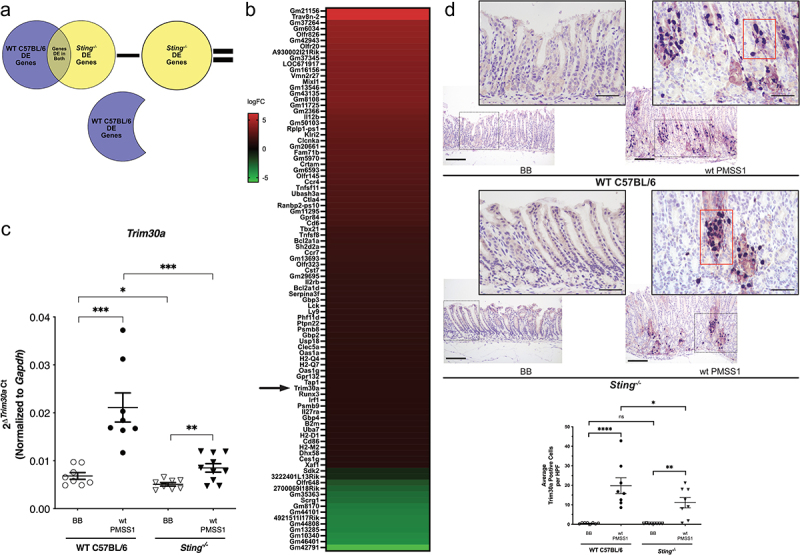
***Trim30a*, a known Sting suppressor, is upregulated by *H. pylori in vivo* in a *Sting*-dependent manner**. (a) Venn diagram representing differentially expressed genes in the RNA-seq dataset of wild-type (WT) C57BL/6 and *Sting^−/−^* mice and schematic of how Sting-dependent genes were determined. (b) Genes that were determined to be dependent on *Sting* are shown by heatmap. Heatmap is displayed as logFC and red indicates upregulation, while green indicates downregulation. *Trim30a* is denoted by the arrow. (c) RT-PCR analysis of *Trim30a* mRNA levels in uninfected and *H. pylori* infected wild-type mice, and uninfected and *H. pylori* infected *Sting^−/−^* mice. Data are represented as relative *Trim30a* gene expression levels normalized to levels of *Gapdh* gene expression. (d) Trim30a was assessed by IHC in wild-type or *Sting^−/−^* mice infected with or without *H. pylori*. Representative images of antral Trim30a IHC are shown at 200x and 400x magnification. Red boxes indicate confirmed co-localization with PMNs. TRIM30a^+^ cells were enumerated in 5 high-powered fields (HPF) from each animal and averaged. Scale bars = 100 µm (200x) and 50 µm (400x). Each data point represents an individual animal (WT BB, n = 8; WT PMSS1, n = 8; *Sting^−/−^* BB, n = 8; *Sting^−/−^* PMSS1, n = 10) from one experiment. Student’s t-tests were used to determine statistical significance between groups. *p < .05, **p < .01, ***p < .001, ****p < .0001, ns = not significant.

We next sought to further refine the identification of mediators of Sting suppression in response to *H. pylori* by filtering the overall differentially expressed gene lists to identify *Sting*-dependent genes. Using the comparison 1 list (Figure 6(b); Supplemental Table 2), any differentially expressed gene that was also identified in comparison 2 was removed (Figure 6(b); Supplemental Table 3), leaving only 92 genes whose differential expression depended on the presence of *Sting* during *H. pylori* infection (Figure 6(b); Figure 8(a); Table 2). The resulting heatmap identified a target that has previously been shown to directly suppress Sting in mice, tripartite motif-containing 30a (Trim30a, Figure 8(b)). TRIM family proteins regulate critical cellular processes such as innate immunity, transcription, and autophagy^58–60^ and can serve as effectors of innate immunity in response to signaling by cytokines such as IFN and tumor necrosis factor alpha (TNFα) and via pattern recognition receptors (PRRs) such as TLR, RIG-I, and STING.^60–63^ Altered levels of *Trim30a* expression were subsequently validated by RT-PCR and significantly higher levels of *Trim30a* were observed in infected wild-type mice compared to *Sting^−/−^* mice (Figure 8(c)). To more precisely define the topography of Trim30a protein expression and clarify localization of the Trim30a in the gastric niche, immunohistochemistry was performed on the murine gastric tissue. Positive staining was observed predominantly in immune cells, such as polymorphonuclear leukocytes (PMNs), and some epithelial cells (Figure 8(d)). When immunohistochemistry was quantitated, *H. pylori* infection induced significantly higher levels of Trim30a expression compared to uninfected controls in both wild-type and *Sting^−/−^* mice. However, infected *Sting^−/−^* mice had significantly lower levels of Trim30a compared to infected wild-type mice (Figure 8(d)), similar to the RNA-seq and RT-PCR data.

**Table 2.** *Sting*-dependent altered gene expression as identified by RNA-seq in *H. pylori* infected C57BL/6 wild-type mice. Up and down regulated genes in C57BL/6 wild-type (WT) *H. pylori* infected mice versus C57BL/6 WT uninfected mice that did not appear in *Sting^−/−^ H. pylori* infected mice versus *Sting^−/−^* uninfected mice. Differential expression analysis was performed on RNA-seq reads. Threshold: log2 fold change≥2 and FDR≤0.05.

Gene	log2 FC
Gm21156	6.147387588
Trav8n-2	6.140669365
Gm37264	4.402125897
Gm6034	4.267729045
Olfr826	4.254651177
Gm42943	4.181455358
Olfr20	4.07809106
A930002I21Rik	3.998040963
Gm37345	3.927665286
LOC671917	3.799049006
Gm16156	3.761233687
Vmn2r27	3.754814719
Mixl1	3.710721859
Gm13546	3.703781216
Gm43135	3.701501962
Gm8108	3.69986602
Gm11725	3.601786512
Gm2366	3.397587749
Il12b	3.230298547
Gm50103	3.086897256
Rplp1-ps1	3.022573925
Klri2	2.957754572
Clcnka	2.79099282
Gm20661	2.774499802
Fam71b	2.772439568
Gm5970	2.712423179
Crtam	2.6003228
Gm6593	2.595129532
Olfr145	2.492549029
Ccr4	2.459354918
Tnfsf11	2.365471002
Ubash3a	2.363320208
Ctla4	2.324780781
Ranbp2-ps10	2.246190307
Gm11295	2.192886019
Gpr84	2.159302175
Cd6	2.065275293
Tbx21	1.838599839
Tnfsf8	1.815705912
Bcl2a1a	1.758799003
Sh2d2a	1.731369243
Ccr7	1.729753456
Gm13693	1.69660395
Olfr323	1.586216282
Cst7	1.559881949
Gm29695	1.485116653
Il2rb	1.469236752
Bcl2a1d	1.462793664
Serpina3f	1.446857109
Gbp3	1.386324632
Lck	1.382141983
Ly9	1.36219576
Phf11d	1.355360699
Ptpn22	1.338582203
Psmb8	1.321643755
Gbp2	1.284264829
Usp18	1.281815099
Clec5a	1.259740759
Oas1a	1.225680027
H2-Q4	1.20655141
H2-Q7	1.200389341
Oas1g	1.197482227
Gpr132	1.165118966
Tap1	1.160037974
Trim30a	1.148855488
Runx3	1.146764074
Irf1	1.136309609
Psmb9	1.125740501
Il27ra	1.123910469
Gbp4	1.0690801
B2m	1.063555331
Uba7	1.060774202
H2-D1	1.060208913
Cd86	1.057121669
H2-M2	1.051679464
Dhx58	1.047740405
Ces1g	1.026262495
Xaf1	1.023047517
Sdk2	−1.054259617
3222401L13Rik	−1.142080967
Olfr648	−2.29420284
2700069I18Rik	−2.658640189
Gm35363	−3.017991589
Scrg1	−3.092599714
Gm8170	−3.460204743
Gm44101	−3.542672499
4921511I17Rik	−3.649908962
Gm44808	−3.650198377
Gm13285	−3.718217905
Gm10340	−3.845960272
Gm46401	−3.969586309
Gm42791	−5.794915899

**Figure 9.**
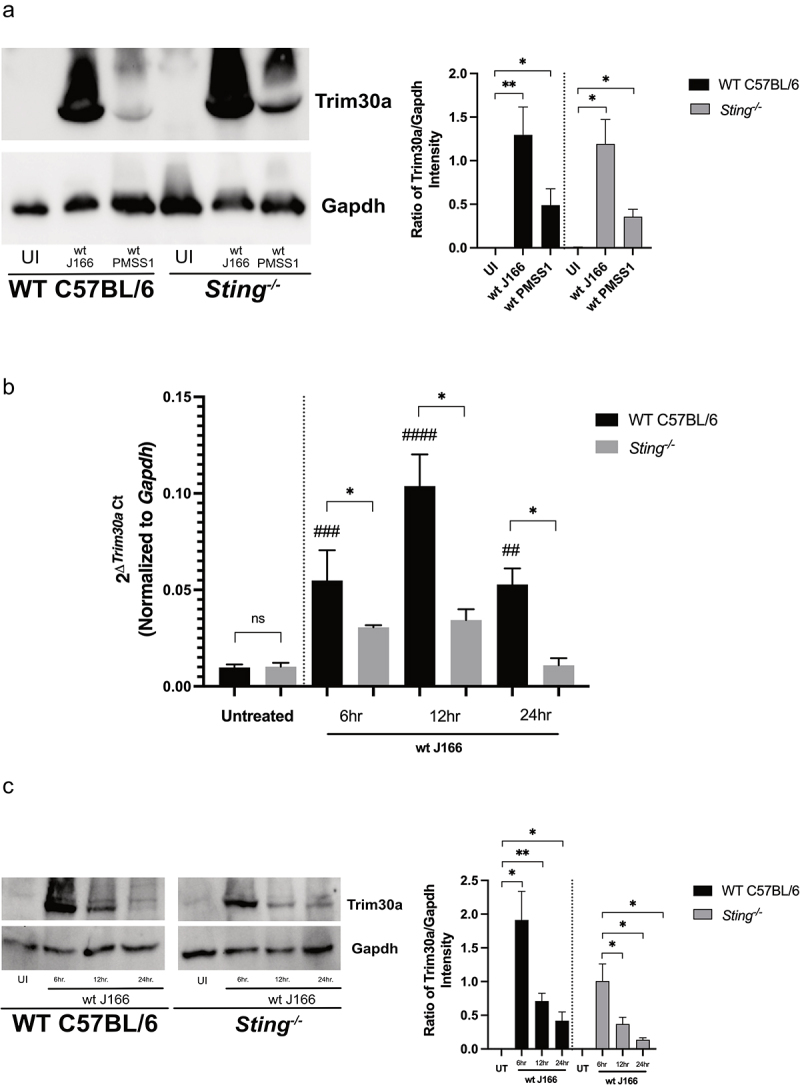
**Trim30a is upregulated by *H. pylori* in a STING-dependent manner**. Murine gastric organoid monolayers or bone marrow derived dendritic cells (BMDC) derived from wild-type (WT) C57BL/6 or *Sting^−/−^* mice were challenged with PBS alone (UI) or wild-type (wt) *H. pylori* strain J166 or PMSS1 at MOI 100:1 for 24 hours. (a) Trim30a was quantified by Western blot analysis in co-cultured murine gastric organoid. Representative Western blots and densitometric analysis normalizing levels of Trim30a to Gapdh. (b) RT-PCR analysis of *Trim30a* mRNA levels in uninfected and *H. pylori*-infected wild-type and *Sting^−/−^* BMDCs. Data are represented as relative gene expression normalized to levels of *Gapdh* gene expression. (c) Trim30a was quantified by Western blot analysis in co-cultured BMDCs. Representative Western blots and densitometric analysis normalizing levels of Trim30a to Gapdh. In each experiment, conditions were tested at least 3 times and student’s t-tests were used to determine statistical significance between groups. *p < .05, **p < .01, ns = not significant. ^##^p < .01, ^###^p < .001 ^####^p < .0001 compared to untreated.

In TLR- and STING-mediated signaling, TRIM30a serves as an important negative feedback regulator that controls excessive inflammatory responses via suppression of type I IFNs production and is expressed in a variety of cell types.^63–65^ Therefore, we next sought to further investigate *ex vivo* and *in vitro* Trim30a expression by *H. pylori* in a cell-specific manner. To examine Trim30a protein expression within gastric epithelial cells, Western blot analysis was performed on co-culture lysates from murine gastric organoid monolayers infected *ex vivo* with *H. pylori* strains J166 and PMSS1 for 24 hours. Significantly increased levels of Trim30a expression were present in both *H. pylori*-infected wild-type and *Sting^−/−^* organoids compared to uninfected cells where no expression was observed (Figure 9(a)). Comparable Trim30a expression patterns were also demonstrated when examined by immunofluorescence in wild-type and *Sting^−/−^* organoids following a 24-hour co-culture with *H. pylori* (Supplemental Figure 6).

Bone marrow derived dendritic cells (BMDC) are also an important source of Trim30a,^63,64^ thus, we next sought to determine if this pathway also functioned similarly within the context of *H. pylori*-induced disease. Therefore, total mRNA was extracted from BMDCs isolated from wild-type or *Sting^−/−^* mice treated with or without *H. pylori* to quantify expression levels of *Trim30a. H. pylori* induced a significant increase in *Trim30a* expression over a 24-hour time course, while *Sting^−/−^* BMDCs exhibited significantly lower levels of *Trim30a* (Figure 9(b)). Western blot analysis on BMDC protein lysates co-cultured with and without *H. pylori* revealed similar findings, with lower Trim30a protein levels observed in infected *Sting^−/−^* BMDCs compared to wild-type BMDCs (Figure 9(c)).

Collectively, in more physiologically relevant *in vivo* murine models, RT-PCR and immunohistochemistry demonstrate that *H. pylori* infection significantly increases *Trim30a* mRNA and protein expression and this response is partially attenuated in *Sting*-deficient mice. In *ex vivo* BMDCs, we observe similar findings, whereby RT-PCR and Western blot analysis demonstrate that *H. pylori* significantly increased *Trim30a* mRNA and protein expression and this response is partially attenuated in BMDCs isolated from *Sting*-deficient mice. It is important to consider however, that isolated peripheral BMDCs may function differently than gastric tissue DCs, which have not been directly assessed in this *ex vivo* model system. Since, in murine gastric organoids, Western blot analysis demonstrated that *H. pylori* significantly increased Trim30a expression, but no differences were observed between gastric organoids isolated from wild-type and *Sting*-deficient animals. These data illustrate the differences between *in vivo* and *ex vivo* models, and we speculate that the differences in Trim30a expression between these models is likely governed by the fact that the murine gastric organoids lack immune components and other elements of the gastric microenvironment that are present in the *in vivo* milieu.

**Figure 10.**
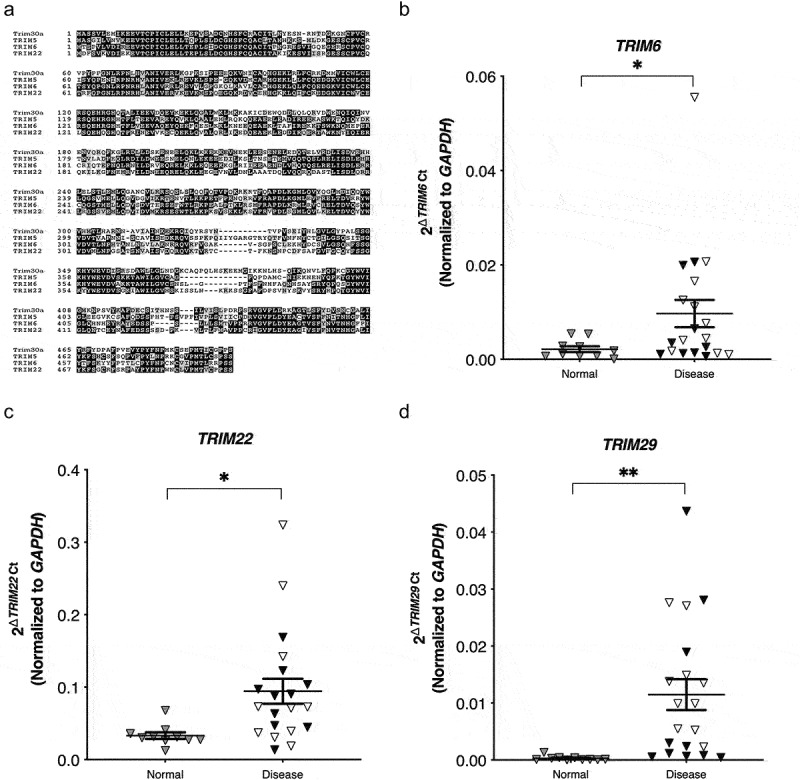
***TRIM6, TRIM22*, and *TRIM29* are upregulated in inflamed or cancerous human clinical gastric specimens**. (a) Multiple sequence alignment of human TRIM30a orthologs to the murine Trim30a protein sequence. The sequence alignment was performed using the T-Coffee program. (b) RT-PCR analysis of *TRIM6, TRIM22*, and *TRIM29* expression in patient samples of normal gastric tissue or samples that harbored inflammation alone (open symbols) or cancer (closed symbols). Data are represented as relative gene expression normalized to levels of *GAPDH* gene expression. Each data point represents an individual patient sample (normal, n = 10; diseased, n = 20). Student’s t-tests were used to determine statistical significance between groups. *p < .05, **p < .01, ****p < .0001.

The mouse specific Trim30a shares greatest homology with specific human TRIMs including TRIM5, TRIM6, and TRIM22 (Figure 10(a)). To extend our findings into human patients, we utilized tissues from a gastric cancer patient cohort to probe for *TRIM30a* human ortholog expression by RT-PCR. Expression of *TRIM6* and *TRIM22*, but not *TRIM5*, was significantly increased in patient samples that harbored inflammation or cancer (Figure 10(b-c); Supplemental Figure 7). Another human TRIM homolog, *TRIM29*, has been directly implicated in STING modulation as well as gastric cancer outcomes;^66–68^ thus, we also analyzed expression levels of *TRIM29* within the patient cohort and demonstrated significantly higher levels of expression in patient samples that harbored inflammation or cancer (Figure 10(d)). These results raise the possibility that TRIMs represent targets induced by *H. pylori* infection, that can suppress STING activation and promote proinflammatory and pro-tumorigenic responses *in vivo*.

## Discussion

Our laboratory has previously demonstrated that *H. pylori* can translocate DNA into host cells activating TLR9^18,31^ but the role of other innate immune nucleic acid sensors during *H. pylori* infection has remained undefined. We now demonstrate using *in vivo, ex vivo*, and *in vitro* data that both STING and RIG-I-associated signaling was suppressed in the presence of viable *H. pylori*. One possible host mechanism for this phenomenon uncovered in our RNA-seq analysis is upregulation of TRIM proteins, which are known innate immune modulators. One such TRIM that was only upregulated in the presence of *Sting* and *H. pylori* was *Trim30a*, a known STING suppressor.^63^ TRIM30a inhibits NF-κB activation induced by TLR signaling, including TLR9, via a K48-linked ubiquitination mechanism that degrades Table 2 and TAB. However, NF-κB activation is required for initial upregulation of TRIM30a expression.^64^ This suggests that TRIM30a may be initially induced by *H. pylori* infection through activation of TLR9 which can then act as a negative regulator of both TLR9 and STING to dampen the subsequent immune response to *H. pylori*. However, to directly assess the role of TRIM30a in the negative regulation of STING, experimental reduction of TRIM30a is needed to more directly assess the effects on both STING and IRF3 activation.

Other *Trim* genes revealed to be upregulated by *H. pylori* (Supplemental Tables 1 and 2) that can modulate innate immune suppression include *Trim40*, which targets the downstream RIG-I regulator MAVS for K48-linked ubiquitination^62^ and *Trim10*, which can suppresses IFN/JAK/STAT signaling pathway through blocking the interaction between IFNAR1 and TYK2 to negatively regulate type I IFN signal transduction.^69^ Our *in vitro* and *ex vivo* work also demonstrated that *H. pylori* could directly mediate innate immune signaling via suppression of IRF3 activation.

Although, we have not directly assessed the role of the *cag* type IV secretion system in this study, it has been well established that the *cag* T4SS translocates the oncoprotein CagA as well as microbial DNA, which can be sensed by innate immune receptors.^18,31^ Another study by Song *et al*.^11^ demonstrated that *H. pylori* infection increases Sting expression and p-Irf3 expression by immunohistochemistry and *Ifnb* gene expression in murine gastric tissue. Although the authors also used C57BL/6 mice, they used a strain of *H. pylori* (SS1) that does not have a functional *cag* type IV secretion system (T4SS) and thus cannot deliver the proinflammatory protein CagA or DNA via the T4SS. We speculate that the lack of a functional T4SS may account of for the differences observed in this model, compared to the current models.

This complex system of innate immune suppression and activation builds on our previous work focused on the duality of TLR9 signaling during *H. pylori* infection and suggests that DNA translocation, induction of TRIM proteins, and suppression of IRF3 activation may be yet another component of a finely tuned rheostat that *H. pylori* utilizes to regulate the initial innate immune response, and ultimately drive long term activation of inflammatory pathways such as those promoted by increased Th17 activation.

In conclusion, this study demonstrates that *H. pylori* actively suppresses innate nucleic acid sensors STING and RIG-I potentially via downregulation of IRF3 activation and induction of TRIM proteins. Additionally, loss of Sting augments acute inflammatory responses to *H. pylori* within the *in vivo* gastric niche. This work lays the foundation for further exploration into the role of *H. pylori-*induced TRIMs in human hosts and suggest that manipulation of TRIMs may represent a novel strategy to prevent or treat pathologic outcomes induced by *H. pylori* infection.

## Materials and methods

### Helicobacter pylori

Wild-type *H. pylori cag*^+^ strains J166, PMSS1,^18,31^ G27,^70^ B128 and 7.13,^71^ were maintained on TSA blood agar plates (Remel). For *in vitro, ex vivo*, and *in vivo* experiments, *H. pylori* was cultured in Brucella broth (Becton Dickinson) supplemented with 10% heat-inactivated FBS with or without 2ʹ3’-cGAMP (30 μg/ml) (Invivogen) overnight at 37°C with 5% CO_2_.

*H. pylori* strains were analyzed for growth as previously described.^72^ Briefly, overnight cultures were sub-cultured in a 96 well flat-bottom plates and incubated in a microaerophilic chamber, as described above. Optical densities (OD) were recorded at 600 nm (BioTek) at 2, 4, 6, 8, 12, and 24 hours. The final OD value was normalized using uninfected media as a negative control.

### Murine models of Sting deficiency

All animal studies were carried out in accordance with the recommendations in the *Guide for the Care and Use of Laboratory Animals* of the NIH. Vanderbilt University Institutional Animal Care and Use Committee approved all protocols. Male and female C57BL/6 wild-type (WT) and *Sting*^−/−^ C57BL/6 mice were purchased from Jackson Laboratories and housed in the Vanderbilt University Animal Care Facilities. Mice were orogastrically challenged with Brucella broth (BB) alone, or with the wild-type *cag^+^ H. pylori* strain PMSS1. Mice were euthanized at 8 weeks post challenge, and gastric tissue was harvested for quantitative culture, histopathology, immunohistochemistry, and RNA isolation and RT-PCR analysis. For quantitative *H. pylori* culture, serial dilutions of homogenized tissue were plated on selective antibiotic TSA-blood agar plates.^17^

### Histopathology

A single pathologist (MBP), blinded to treatment groups, scored indices of inflammation and injury as described previously.^17,73^ Specifically, the following variables were graded on a 0 to 3 scale (0, none; 1, mild; 2, moderate; 3, severe) in the gastric antrum and body: acute inflammation (polymorphonuclear cell infiltration) and chronic inflammation (mononuclear cell infiltration independent of lymphoid follicles); thus a maximum inflammation score of 12 was possible for each animal.

### Immunohistochemistry

Murine gastric tissue sections were stained with anti-TMEM173/STING antibody #19851-1-AP (Proteintech; 1:100), anti-MPO antibody #PP023AA (Biocare Medical; Ready-to-use), anti-CD68 antibody #PM033AA (Biocare Medical; Ready-to-use), anti-CD45 antibody #10558 (Abcam; 1:4000), anti-CD11c rabbit antibody #97585 (Cell Signaling; 1:2400), and anti-RIG-I rabbit antibody #700366 (Invitrogen;1:100). Anti-CD3 antibody #Ab16669 (Abcam; 1:250) and anti-TRIM30 antibody #NBP2-41087 (Novus Biologicals; 10 μg/ml) were used to stain gastric tissue sections by the Vanderbilt University Medical Center Translational Pathology Shared Resource (TPSR). A single pathologist (MBP), blinded to treatment groups, evaluated and scored all immunohistochemistry (IHC). Trim30a, MPO, CD3, CD68, CD45, and CD11c were evaluated by quantifying positive cells in 5 HPFs (400x) with the highest counts in each mouse. Sting and RigI staining were evaluated by assessing the percentage of positive epithelial cells and grading the intensity of staining in epithelial cells semi-quantitatively, as previously described.^74^

### Cell culture

HEK293 hSTING-R232 cells (STING+), HEK293 Null (STING Parental) cells, HEK-Lucia RIG-I cells (RIG-I+), and HEK-Lucia Null cells (RIG-I Parental) (Invivogen) were grown in DMEM (ThermoFisher) supplemented with 10% FBS and 100 μg/mL Zeocin (Invivogen). STING+ and RIG-I+ cell media was supplemented with an additional selective antibiotic, Blasticidin (Invivogen) at 10 μg/mL. AGS human gastric epithelial cells (ATCC CRL-1739) were grown in RPMI 1640 (ThermoFisher) with 10% FBS. All cell lines were maintained at 37°C with 5% CO_2_.

Human-derived gastric epithelial monolayers^75^ and mouse primary gastric epithelial cell monolayers^17^ were generated as previously reported.^50^ Briefly, human fundus was collected during sleeve gastrectomies according to a University of Cincinnati Institutional Review Board-approved protocol (#2015-4869), after informed consent was obtained. For murine organoids, gastric glands were harvested from uninfected wild-type or *Sting^−/−^* mice at least 8 weeks of age. Gastric tissue was washed and digested, and isolated glands were incubated in Matrigel (Corning).^76^ Primary gastric organoids were then converted to 2D epithelial cell monolayers following Matrigel removal and 3D gastric organoids were plated on collagen-coated plates.

Bone marrow-derived dendritic cells (BMDC) were generated from bone marrow obtained from femurs of wild-type and *Sting^−/−^* mice. Briefly, marrows were treated with red blood cell lysis buffer (KD Medical) and washed with PBS and recovered white blood cells were plated in advanced DMEM media (Gibco) supplemented with 20% FBS and 40 ng/mL each of GM-CSF and IL-4 (Peprotech) for 6 days at 37°C and 5% CO_2_ for differentiation.

### Ex vivo and in vitro infections

Primary 2D gastric organoid monolayers were co-cultured with or without 2ʹ3’-cGAMP (30 μg/ml) and *H. pylori* strains J166 or PMSS1 at a multiplicity of infection (MOI) of 100:1 for 6 or 24 hours. BMDCs were co-cultured with wild-type *H. pylori* strain J166 at a multiplicity of infection (MOI) of 10:1 or ODN-1826 (6 μg/ml) for 6, 12, or 24 hours. RNA and protein were then isolated from co-culture samples for RT-PCR and Western blot analysis, respectively.

### STING reporter assay

HEK293 hSTING-R232 cells (STING+) and HEK293 Null1 (Parental) cells were seeded in 96-well plates (Corning) at 50,000 cells per well in DMEM without antibiotics and challenged with either viable *H. pylori* (MOI 100:1), sterile PBS, and/or 2ʹ3-cGAMP (30 μg/ml) at 37°C with 5% CO_2_ for 24 hours. Supernatants were then added to QUANTI-Blue™ solution (Invivogen) and plates were analyzed by spectrophotometer (Bitoek) at 650 nm. All experiments were performed in duplicate and repeated at least three times. Data are expressed as fold over uninfected control.

### RIG-I reporter assay

HEK-Lucia RIG-I cells (RIG-I+), and HEK-Lucia Null cells (RIG-I Parental) cells were seeded in 96-well plates (Corning) at 50,000 cells per well in DMEM without antibiotics and challenged with either viable *H. pylori* (MOI 100:1), sterile PBS, and/or 3php-RNA (5000 ng/ml) at 37°C with 5% CO_2_ for 24 hours. Supernatants were then added to QUANTI-Luc™ solution (Invivogen) and plates were analyzed by luminometer (Bitoek). Experiments were performed in triplicate, and samples were run in duplicate within each experiment. Data are expressed as fold over uninfected control.

### Cell viability assay

The effect of *H. pylori* and/or agonists on reporter cell viability was assessed in STING+, RIG-I+, and their respective parental cells using the CellTiter-Blue assay (Promega), according to the manufacturer’s instructions. In brief, following co-culture, STING or RIG-I reporter cells were washed with PBS containing gentamycin (250 μg/ml) and hygromycin (500 μg/ml), followed by incubation with DMEM media containing gentamycin (250 μg/ml) and hygromycin (500 μg/ml). After a 30-minute incubation at 37°C to eliminate residual viable *H. pylori*, CellTiter-Blue reagent was added. Samples were incubated for 1 hour at 37°C and fluorescence was measured (485_Ex_/516_Em_) using a fluorescent imaging plate reader (Biotek).

### Real-time PCR

RNA was extracted using the RNAeasy Mini Kit (Qiagen) for all sample types, according to the manufacturer’s instructions. cDNA was synthesized using High-Capacity cDNA Reverse Transcription Kit (ThermoFisher) and quantitative real-time PCR was performed using Power SYBR Green Master Mix (ThermoFisher) for human samples and TaqMan™ Universal Master Mix II (ThermoFisher) for murine samples, with gene-specific primers (Table 3).

**Table 3.** List of primers and assays used for RT-PCR analyses.

Human RT-PCR primers	
Primer Name	DNA sequence
*CXCL10*-F	5’-GCAGTTAGCAAGGAAAGGTCTAA-3’
*CXCL10*-R	5’-ATGTAGGGAAGTGATGGGAGAG-3’
*GAPDH*-F	5’-AGCCTCAAGATCATCAGCAATG-3’
*GAPDH*-R	5’-GGGTGCTAAGCAGTTGGTGG-3’
*MX1*-F	5’-GTGGCTGAGAACAACCTGTG-3’
*MX1*-R	5’-GGCATCTGGTCACGATCCC-3’
*TRIM5*-F	5’-GCTCTCCGAAACCACAGATAA-3’
*TRIM5*-R	5’-CCCAGGATGCCAGTACAATAA-3’
*TRIM6*-F	5’-GGAGGATGGGAAGGTCATTT-3’
*TRIM6*-R	5’-CCTGAAACTTCTCCTGGTACTC-3’
*TRIM22*-F	5’-TGGAAGATCGAGAGACAGAAGA-3’
*TRIM22*-R	5’-CCAGGTTATCCAGCACATTCA-3’
*TRIM29*-F	5’-GACCTGCATCTGCTACCTTT-3’
*TRIM29*-R	5’-ACAGCTCCGTCTCCTTCT-3’
Mouse Integrated DNA Technologies (IDT) PrimeTime™ qPCR probe assays	
Gene	**Assay ID**
*Cxcl10*	Mm.PT.58.43575827
*Gapdh*	Mm.PT.39a.1
*Icos*	Mm.PT.58.6938712
*Ifng*	Mm.PT.58.41769240
*Il1b*	Mm.PT.58.41616450
*Il6*	Mm.PT.58.10005566
*Il12b*	Mm.PT.58.12409997
*Il17a*	Mm.PT.58.6531092
*Il17f*	Mm.PT.58.9739903
*Il23*	Mm.PT.58.41340226
*Irf4*	Mm.PT.58.31041855
*Mx1*	Mm.PT.58.12101853
*Trim30a*	Mm.PT.56a.43098591

### Western blot analysis

AGS cells, human, and murine organoids co-cultured with *H. pylori* were and protein lysates were separated using 6% (AGS cells) or 10% (organoids) SDS PAGE mini gels, transferred to PVDF membranes, and membranes were blocked with BSA or milk as denoted. For detection of proteins, membranes were incubated overnight with anti-CagA rabbit antibody (Austral Biologicals; 1:5000, BSA), anti-pY99 antibody mouse antibody (Santa Cruz; 1:5000, BSA), anti-phospho-IRF-3 rabbit antibody #29047 (Cell Signaling Technology; 1:1000, BSA), anti-IRF-3 rabbit antibody #11904 (Cell Signaling Technology; 1:1000, BSA), anti-phospho-TBK1/NAK rabbit antibody #5483 (Cell Signaling Technology; 1:1000, BSA), anti-TBK1/NAK rabbit antibody #38066 (Cell Signaling Technology; 1:1000, BSA), anti-LC3A/B rabbit antibody #4108 (Cell Signaling Technology; 1:1000, BSA), anti-TMEM173/STING rabbit antibody #19851-1-AP (Proteintech; 1:1000, BSA), anti-GAPDH mouse antibody #MAB374 (Millipore Sigma; 1:5000, BSA), or anti-TRIM30 rabbit antibody #NBP2-41087 (Novus Biologicals; 1:1000, milk). An anti-rabbit or anti-mouse HRP-conjugated secondary antibody (Promega; 1:10000) was then incubated with membranes for 1 hour. Protein intensities were quantified using ImageJ software (NIH).

### Trim30a immunofluorescence staining

Monolayers of primary gastric epithelial cells derived from C57BL/6 wild-type and *Sting^−/−^* mice were infected for 24 hours with *H. pylori* strains J166 or PMSS1. After infection, monolayers were subjected to immunofluorescence staining as previously described.^17,50^ Briefly, cells were fixed with 10% neutral-buffered formalin (Azer Scientific), permeabilized with Triton X-100 (Promega), and then blocked with Dako Protein Block Solution (Agilent) for 1 hour. Samples were incubated with anti-TRIM30 rabbit antibody #NBP2-41087 (Novus Biologicals; 1:50) overnight at 4°C before detection with Alexa-fluor secondary antibody (Invitrogen). Nuclei were detected using Hoescht (Invitrogen). Slides were mounted using ProLong Glass (Invitrogen), and images were acquired in an Olympus FV-1000 confocal microscope. Experiments were performed in part through the use of the Vanderbilt Cell Imaging Shared Resource (CISR).

### RNA sequencing and analysis

Total RNA from wild-type C57BL/6 and *Sting^−/−^* mice was processed using a NEBNext® Ultra™ II RNA Library Prep sample prep kit following the manufacturer’s instructions (New England Biosciences) and evaluated on a Qubit 3.0 fluorometer and an Agilent 2100 Bioanalyzer to quantitate concentration and fragment size distribution prior to sequencing using the NovaSeq 6000 sequencer (Illumina). Sequencing was performed using a S4 flow cell with a PE 150 kit. Individually barcoded libraries were then pooled at an equal molar ratio and sequenced at 2 × 150 bp/read. Approximately 48 million paired-end sequence reads per sample (mean ± SD = 48.3856 ± 7.655 million; n = 34) were generated. Sample quality was assessed via FastQC software. The data were analyzed using the Dragen Software Version: 3.6.3 aligning the data to the mm10 reference genome. The Vanderbilt Technologies for Advanced Genomics (VANTAGE) core facility prepared the RNA library, assessed library quality, and performed sequencing. Differential expression analyses were performed at baseline (uninfected WT C57BL/6 versus *Sting^−/−^*) and based on the following 2 major comparisons: wild-type C57BL/6 infected versus wild-type C57BL/6 Brucella broth control, and *Sting^−/−^* infected versus *Sting^−/−^* Brucella broth control. Filters used to identify differential expression were an adjusted p-value <0.05 and an absolute log2 fold change >1. Venn diagrams were created (Venny 2.1 software) using these comparisons. Ingenuity Pathway Analysis software (Qiagen) was used to link differentially expressed genes in the dataset to particular biological functions and pathways.

### Human clinical specimens

Snap frozen de-identified human gastric tissue samples were acquired from the Cooperative Human Tissue Network (CHTN). Normal gastric tissue, or gastric tissues harboring either gastritis alone or gastric adenocarcinoma were disrupted and homogenized using ZR BashingBead Lysis tubes (Zymo Research) prior to RNA extraction. The protocol was approved by the Vanderbilt University Medical Center Institutional Review Board (#210729).

### Statistics

The student’s t-test was used for two group comparisons, while one-way analysis of variance (ANOVA) with Bonferroni correction was used for multiple group comparisons. Data were plotted and analyzed using Prism 6.0 (GraphPad). Statistical significance was set at a two-tailed p-value of <0.05. In all figures, means ± standard errors of the mean are shown.

### Ethics of experimentation

All animal and human studies were conducted in accordance with the Declaration of Helsinki principles and have been approved by the Vanderbilt University Medical Center Institutional Animal Care and Use Committee (IACUC) and the Vanderbilt University Medical Center Institutional Review Board (IRB), respectively. Human gastric tissue samples used for RT-PCR were acquired from the Cooperative Human Tissue Network and these analyses were approved under IRB #210729 under the category of a non-human subject study.

## Supplementary Material

Supplemental Material

## Data Availability

The authors confirm that the data supporting the findings of this study are available within the article and its supplemental materials.
