# Supplementary material for: Helicobacter pylori actively suppresses innate immune nucleic acid receptors
Source: Gut Microbes. 2022 Jul 29;14(1):2105102. doi: 10.1080/19490976.2022.2105102 (PMC9341374; doi:10.1080/19490976.2022.2105102)
Supplement: Supplemental Material [file KGMI_A_2105102_SM1864.zip › Revised Supplemental Materials_FINAL.docx]

**Supplemental Figure Legends**

**Supplemental Figure 1. Cell viability of STING+ and parental cells.** STING+ or parental cells were challenged with PBS alone (UI), Triton X-100, STING agonist 2’3’-cGAMP and/or wild-type (wt) *cag^+^* *H. pylori* strain J166 at MOI 100:1 for 4-24 hours. CellTiter-Blue^®^ assay was performed to determine cell viability. Experiments were performed in triplicate, and samples were run in duplicate within each experiment. Data are shown as fold percent cell viability, relative to positive control (UI) and negative control (TritonX-100). ANOVA with Bonferroni correction was used to determine statistical significance among groups. ****p<0.0001.

**Supplemental Figure 2. STING agonist 2’3’-cGAMP does not directly affect *H. pylori* growth or *cag* T4SS function.** (A) Wild-type (wt) *cag^+^* *H. pylori* strain J166 was grown in Brucella broth supplemented with 10% fetal bovine serum (FBS) alone or supplemented with 5, 10, or 30µg/ml 2’3’-cGAMP for 2, 4, 6, 12, 18, and 24 hours in the absence of eukaryotic cells. Bacterial growth was assessed by spectrophotometric reading at OD600. (B) Wild-type *cag^+^* *H. pylori* strain J166 or isogenic *cagE^-^* mutant (negative control) were grown overnight in either the presence or absence of 2’3’-cGAMP, in the absence of eukaryotic cells. AGS cells were subsequently co-cultured with *H. pylori* at MOI 100:1 for 4 hours. CagA expression (CagA) and translocation (p-Tyr) were determined by Western blot analysis. Representative Western blots and densitometric analysis normalizing levels of phosphorylated CagA to total CagA are shown. GAPDH is shown as a loading control. Experiments were performed in triplicate and ANOVA with Bonferroni correction (A) or student’s t-tests (B) were used to determine statistical significance between groups. ns=not significant.

**Supplemental Figure 3. Cell viability of RIG-I+ and parental cells.** RIG-I+ or parental cells were challenged with PBS alone (UI), Triton X-100, Lipofectamine 2000, 3p-hpRNA and/or and/or *H. pylori* strain J166. CellTiter-Blue^®^ assay was performed to determine cell viability. Experiments were performed in triplicate, and samples were run in duplicate within each experiment. Data are shown as fold percent cell viability normalized to Lipofectamine 2000 control, relative to positive control (UI) and negative control (TritonX-100). ANOVA with Bonferroni correction was used to determine statistical significance among groups. None of the comparisons were statistically significant.

**Supplemental Figure 4. *H. pylori* infection significantly augments chronic inflammation independent of *Sting* deficiency.** Wild-type C57BL/6 (WT) and *Sting^−/−^* mice were challenged with Brucella broth (BB) or wild-type *H. pylori* strain PMSS1 for 8 weeks. Chronic inflammation was assessed and scored in the antrum and corpus by a pathologist blinded to treatment groups. Histologic parameters were scored according to the Sydney System.^76^ Each data point represents an individual animal (WT BB, n=8; WT PMSS1, n=8; *Sting^-/-^* BB, n=8; *Sting^-/-^* PMSS1, n=10) from one experiment. Student’s t-tests were used to determine statistical significance between groups. ***p<0.001, ****p<0.0001, ns=not significant.

**Supplemental Figure 5. Levels of macrophages, T-cells, and B-cells *in vivo* are increased with in the presence of *H. pylori*, regardless of *Sting* status.** Levels of (A) CD68 (B) CD3 and (C) CD45 positive cells in wild-type or *Sting^−/−^* mice infected with or without *H. pylori*. Each data point represents an individual animal (WT BB, n=8; WT PMSS1, n=8; *Sting^-/-^* BB, n=8; *Sting^-/-^* PMSS1, n=10) from one experiment. Positive cells were enumerated in 5 high-powered fields from each animal and averaged. Student’s t-tests were used to determine statistical significance between groups. ****p<0.0001, ns=not significant.

**Supplemental Figure 6. *H. pylori* increases Trim30a expression in murine gastric organoids.** Murine gastric organoid monolayers derived from wild-type or *Sting^-/-^* mice were challenged with PBS alone (UI), *H. pylori* wild-type strain J166 or PMSS1 at MOI 100:1 for 24 hours. Trim30a was assessed by immunofluorescence. Trim30a is shown in green and nuclei (DAPI) are shown in blue. Experiments were performed in triplicate and representative images of Trim30a IF are shown at 40x magnification. Scale bars = 100µm.

**Supplemental Figure 7. *TRIM5* expression in human clinical stomach specimens.** RT-PCR analysis of *TRIM5* expression in patient samples with normal gastric tissue or samples that harbored inflammation alone (open symbols) or cancer (closed symbols). Data are represented as relative *TRIM5* gene expression levels normalized to levels of *GAPDH* gene expression. Each data point represents an individual patient sample (normal, n=10; diseased, n=20). Student’s t-tests were used to determine statistical significance between groups. ns=not significant.
