# Supplementary figures and images for: Helicobacter pylori actively suppresses innate immune nucleic acid receptors
Source: Gut Microbes. 2022 Jul 29;14(1):2105102. doi: 10.1080/19490976.2022.2105102 (PMC9341374; doi:10.1080/19490976.2022.2105102)

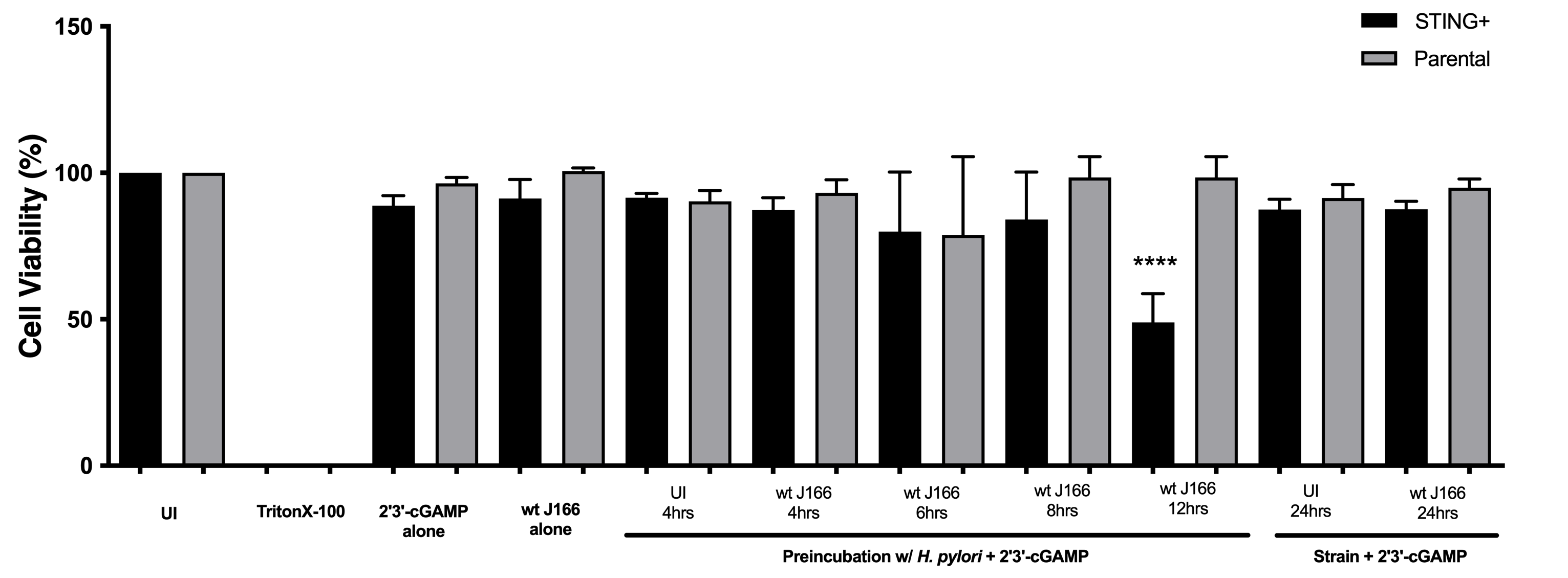

Supplement: Supplemental Material [file KGMI_A_2105102_SM1864.zip › Supplemental Figure 1.tiff]

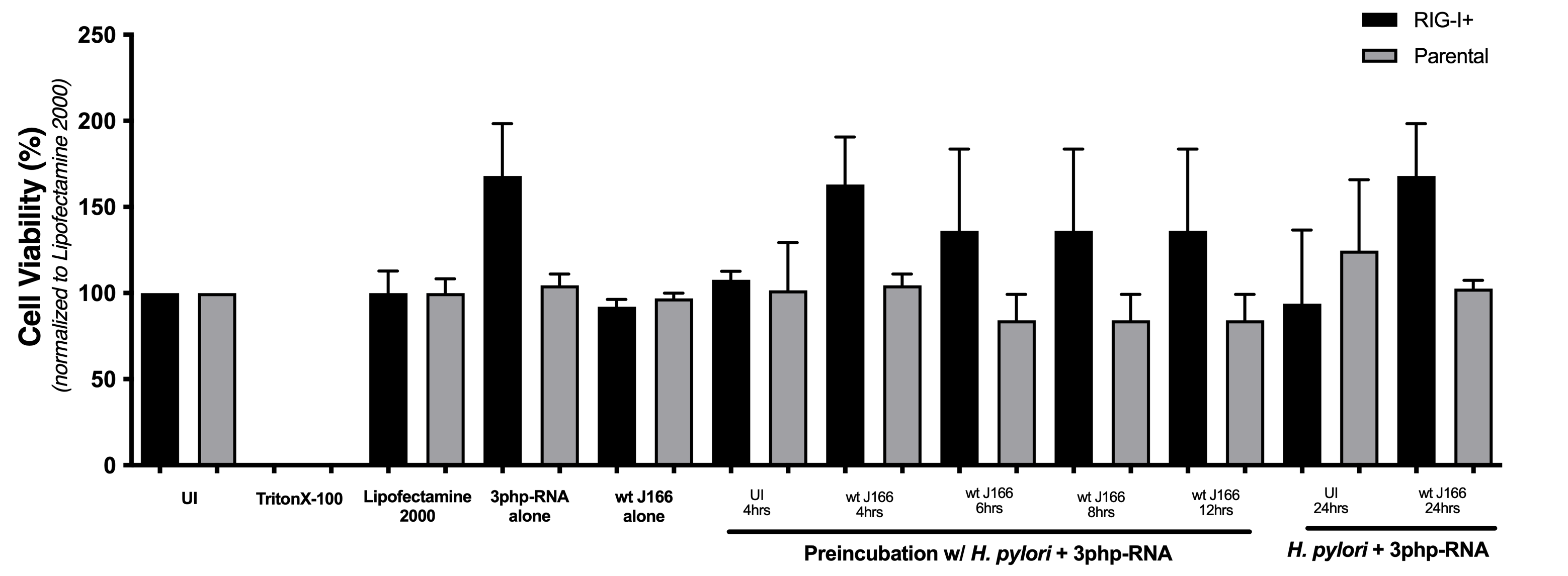

Supplement: Supplemental Material [file KGMI_A_2105102_SM1864.zip › Supplemental Figure 3.tiff]

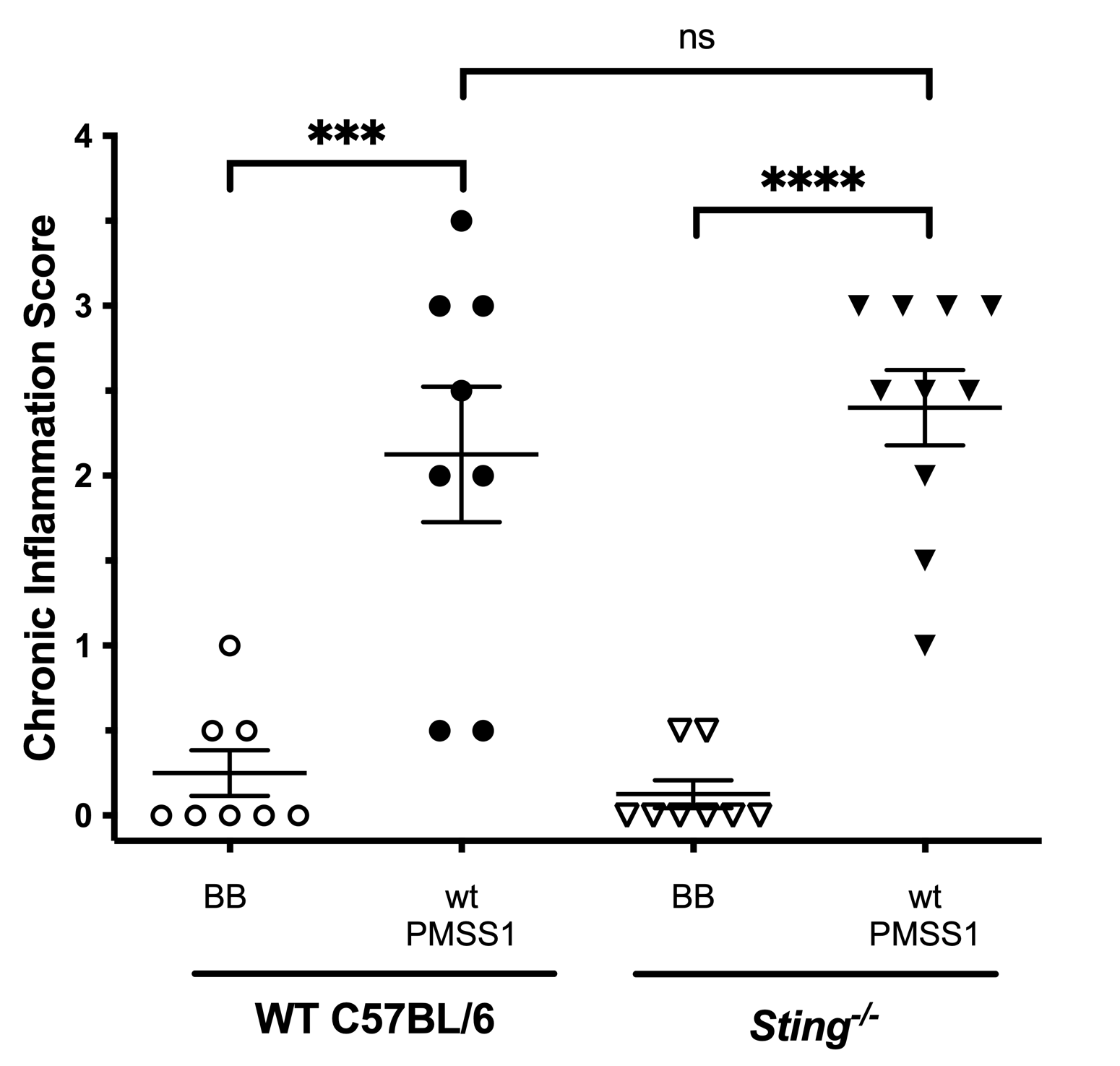

Supplement: Supplemental Material [file KGMI_A_2105102_SM1864.zip › Supplemental Figure 4_revised.tiff]

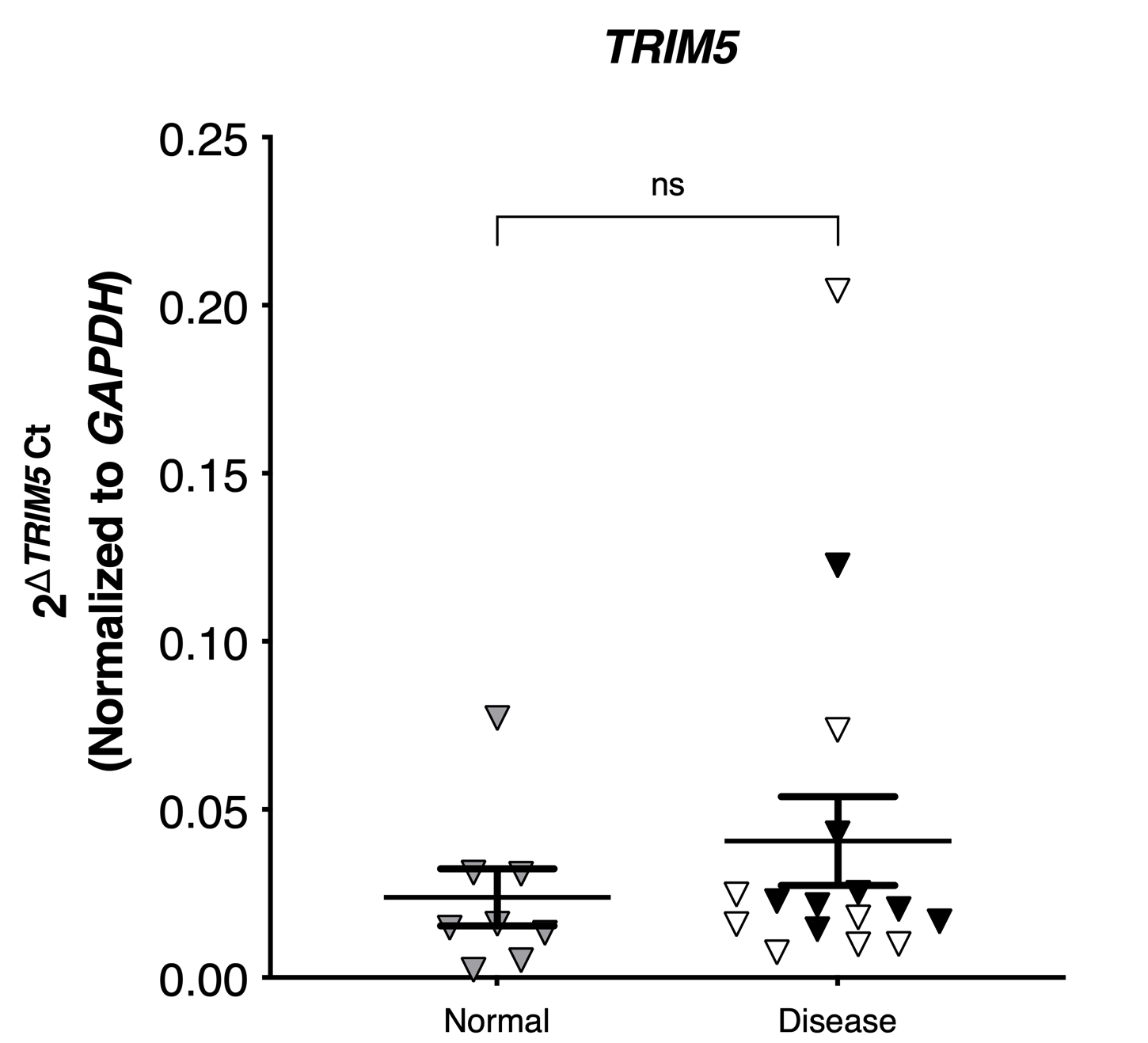

Supplement: Supplemental Material [file KGMI_A_2105102_SM1864.zip › Supplemental Figure 7_revised.tiff]
